# Adaptive Responses of Hormones to Nitrogen Deficiency in *Citrus sinensis* Leaves and Roots

**DOI:** 10.3390/plants13141925

**Published:** 2024-07-12

**Authors:** Dan Hua, Rong-Yu Rao, Wen-Shu Chen, Hui Yang, Qian Shen, Ning-Wei Lai, Lin-Tong Yang, Jiuxin Guo, Zeng-Rong Huang, Li-Song Chen

**Affiliations:** College of Resources and Environment, Fujian Agriculture and Forestry University, Fuzhou 350002, China; 1210807008@fafu.edu.cn (D.H.); 1220807012@fafu.edu.cn (R.-Y.R.); 1220807020@fafu.edu.cn (W.-S.C.); 52308031045@fafu.edu.cn (H.Y.); 52308031061@fafu.edu.cn (Q.S.); lainingwei1109@fafu.edu.cn (N.-W.L.); talstoy@fafu.edu.cn (L.-T.Y.); jxguo@fafu.edu.cn (J.G.); huangzengrong@fafu.edu.cn (Z.-R.H.)

**Keywords:** abscisic acid, *Citrus sinensis*, cytokinin, gibberellin, indole-3-acetic acid, jasmonic acid, root system architecture

## Abstract

Some citrus orchards in China often experience nitrogen (N) deficiency. For the first time, targeted metabolomics was used to examine N-deficient effects on hormones in sweet orange (*Citrus sinensis* (L.) Osbeck cv. Xuegan) leaves and roots. The purpose was to validate the hypothesis that hormones play a role in N deficiency tolerance by regulating root/shoot dry weight ratio (R/S), root system architecture (RSA), and leaf and root senescence. N deficiency-induced decreases in gibberellins and indole-3-acetic acid (IAA) levels and increases in cis(+)-12-oxophytodienoic acid (OPDA) levels, ethylene production, and salicylic acid (SA) biosynthesis might contribute to reduced growth and accelerated senescence in leaves. The increased ethylene formation in N-deficient leaves might be caused by increased 1-aminocyclopropanecarboxylic acid and OPDA and decreased abscisic acid (ABA). N deficiency increased R/S, altered RSA, and delayed root senescence by lowering cytokinins, jasmonic acid, OPDA, and ABA levels and ethylene and SA biosynthesis, increasing 5-deoxystrigol levels, and maintaining IAA and gibberellin homeostasis. The unchanged IAA concentration in N-deficient roots involved increased leaf-to-root IAA transport. The different responses of leaf and root hormones to N deficiency might be involved in the regulation of R/S, RSA, and leaf and root senescence, thus improving N use efficiency, N remobilization efficiency, and the ability to acquire N, and hence conferring N deficiency tolerance.

## 1. Introduction

Nitrogen (N) is one of the key factors affecting crop growth and yield [1,2]. Some citrus orchards often experience N deficiency. In the main citrus producing areas of China, 59.8% of the orchards were deficient in soil-available N [3].

Evidence shows that N deficiency-induced increases in root/shoot dry weight (DW) ratio (R/S), alterations in root system architecture (RSA), and delaying in root senescence play a role in plant N deficiency tolerance [4,5,6]. Hormones are involved in diverse aspects of plant growth and development [7]. Therefore, these changes caused by N deficiency should be related to the changes in hormones in roots and shoots (leaves) [8,9,10]. In maize, Tian et al. [11] observed that N supply decreased indole-3-acetic acid (IAA) concentration in roots and that the inhibitory impact of high N on primary root growth could be restored by the application of IAA. Further study indicated that low N increased shoot-to-root IAA transport, thereby enhancing IAA concentration in root tips which promoted root elongation through auxin (AUX)-induced acid growth and AUX-regulated targeting of the rapamycin pathway in maize, and that when shoot-to-root IAA transport was hindered, the increase in root elongation induced by low N was greatly repressed [12].

The application of ethylene (ETH) can reduce root growth. N deprivation lowers ETH formation in maize roots [9]. Cytokinins (CKs) repress root growth and promote shoot growth [13]. High nitrate may enhance CK accumulation in roots, which can increase ETH biosynthesis and hinder AUX action, thereby lowering root elongation [9]. However, in pumpkins, Mardanov et al. [14] observed that N-starved roots had increased levels of CKs, and reduced levels of IAA and abscisic acid (ABA), and N-starved shoots had increased concentrations of ABA and reduced concentrations of CKs and IAA, concluding that N starvation induced an increase in root growth and a decrease in shoot growth was related to a high CK/ABA ratio in roots and a low CK/ABA ratio in shoots.

The first characteristic physiological impact of jasmonic acid (JA) is to inhibit plant growth [15]. ABA is regarded as a growth inhibitor [16]. High concentrations of salicylic acid (SA) have an inhibitory effect on plant growth [17]. In tomatoes, N deficiency increased the levels of ABA, IAA, SA, jasmonoyl-L-isoleucine (JA-ILE), and JA in the xylem sap, and decreased leaf area and plant biomass [18].

Camut et al. [1] showed that nitrate promoted leaf sheath elongation in wheat, and stem elongation and shoot branching in *Arabidopsis* through improving gibberellin (GA) biosynthesis and accumulation. N deficiency lowered the concentrations of GA_1_, GA_4_, and GA_3_ in maize roots [19], and increased the concentration of GA_3_ in wheat roots [20].

*Arabidopsis* strigolactone (SL)-deficient mutants had reduced length of the primary roots and number of cells in the meristem zone than the wild-type plants. A low concentration of the SL analog GR24 increased the two parameters in the SL-deficient mutants and wild-type plants [9]. Growing evidence shows that the elevated accumulation and exudation of SLs in N-deficient roots is a key adaption, because SLs may enhance N uptake through altering RSA [21,22].

Leaf senescence plays a key role in the economy and recycling of N in plants [23]. Studies suggested that N deficiency-induced leaf senescence was an adaptive response to N deficiency by enhancing N remobilization from senescing to N-demanding tissues [24,25]. ABA, SA, ETH, JA, and SLs can promote leaf senescence, while CKs can delay leaf senescence [26]. The roles of AUXs and GAs in leaf senescence are elusive [27,28]. Zakari et al. [29] indicated that the elevated levels of ABA and reactive oxygen species in N-deficient rice leaves was the main cause for accelerating leaf senescence and that the application of ABA accelerated the senescence of rice leaves. However, Oka et al. [23] demonstrated that ABA accumulated in N-limited cucumber shoots and that the application of ABA inhibited leaf senescence of cucumber plants grown under N limitation. N starvation reduced ABA levels in tomato leaves [30] and did not alter ABA levels in *Arabidopsis* shoots [5]. Application of *trans*-zeatin (tZ) repressed N deficiency-induced senescence in wheat leaves [31]. N deficiency increased ETH evolution in mustard leaves [32], SA levels in *Arabidopsis* shoots [5], (+)-5-deoxystrigol (5DS) levels in grape leaves [33], and wound-induced accumulation of JA in maize leaves [34]. However, N starvation decreased JA concentrations in *Arabidopsis* shoots [5] and SA and OPDA levels in tomato leaves [30].

Although some researchers have investigated plant hormone responses to N deficiency, most of the work has focused on model plants and herbs, and the results are inconsistent. To date, very little is known about the impacts of N deficiency on hormones in woody horticultural plants [9]. Additionally, most researchers only examined the effects of N deficiency on one or several hormones in roots or leaves [8,32,35,36], which may miss a comprehensive investigation on the contribution of hormones in the N deficiency tolerance of plants. To our knowledge, data on the effects of N deficiency on hormone metabolomics in leaves and roots are rare.

Recent work from our laboratory showed that N deficiency increased R/S and root-surface-per-unit volume, delayed root senescence, and accelerated leaf senescence in sweet orange (*Citrus sinensis* (L.) Osbeck cv. Xuegan) seedlings, thereby improving N remobilization efficiency (NRE), N use efficiency (NUE), and the ability to acquire N, and hence conferring N deficiency tolerance [2,4,6,25,37]. Based on the previous studies, we used targeted metabolomics to examine N-deficient effects on hormones in ‘Xuegan’ leaves and roots. Also, we used RNA-Seq to investigate N-deficiency-responsive genes related to hormone metabolism in leaves and roots. The aims were (a) to validate the hypothesis that hormones are involved in citrus N deficiency tolerance by regulating R/S, RSA, and leaf and root senescence and (b) to reveal the differences in hormone changes caused by N deficiency in leaves and roots.

## 2. Results

### 2.1. Effects of N Supply on Hormones and Related Metabolites (HRMs) in Leaves and Roots

**Figure 1:**
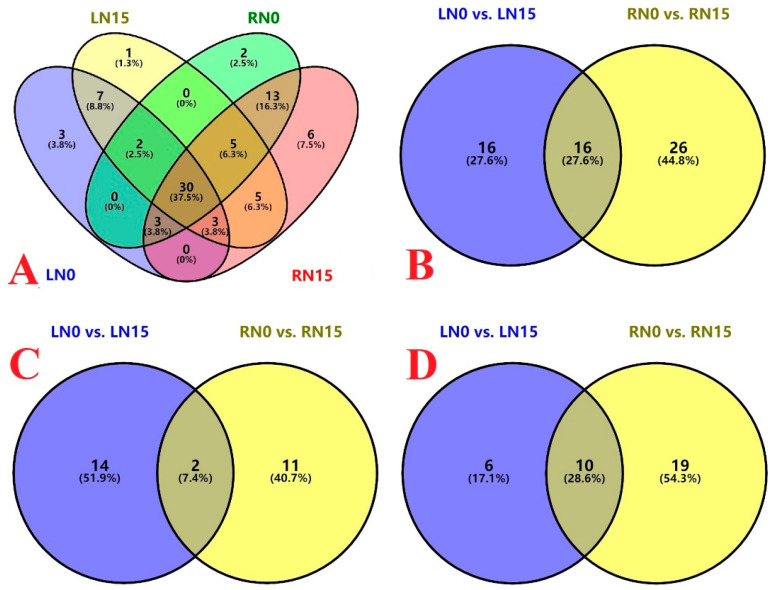
Venn diagrams of HRMs detected in LN0, LN15, RN0, and RN15 (**A**), and total differentially abundant HRMs (**B**), upregulated HRMs (**C**), and downregulated HRMs (**D**) in LN0 vs. LN15 and RN0 vs. RN15.

We tested 88 HRMs in leaves and roots (Table S1), 80 of which were detected in leaves and/or roots, including 48, 53, 55, and 65 HRMs in the leaves of 0 mM N-treated seedlings (LN0), leaves of 15 mM N-treated seedlings (LN15), roots of 0 mM N-treated seedlings (RN0), and roots of 15 mM N-treated seedlings (RN15), respectively. Among the 80 HRMs, three, one, two, and six HRMs were detected only in LN0, LN15, RN0, and RN15, respectively. Thirty HRMs were simultaneously detected among the four samples (Table 1 and Figure 1A).

We detected 59 HRMs in LN0 and/or LN15, including 2 ABA and its metabolic products (hereafter referred to as abscisates (ABAs)), 17 AUXs, 22 CKs, ACC, 4 GAs, 9 JAs, 2 SLs, and 2 SAs. Isoprenoid and aromatic CKs are present in plants, with the former being more common and having higher concentrations than the latter [38]. Among these 22 CKs, 14 belong to isoprenoid CKs and 8 belong to aromatic CKs. A total of 6 and 11 HRMs were detected only in LN0 and LN15, respectively. There were 42 common HRMs between the two (Table 1 and Figure 1A).

In leaves, 0 mM N treatment (N0) increased TRP, TRA, IAA-Glu, IAA-VAL, and ILA concentrations relative to 15 mM N treatment (N15), and had no significant impacts on MEIAA, IAA-Asp, IAA-Gly, IAA-Trp, IAA-Phe-Me, IAA-Leu-Me, and ICAld concentrations. IAA, IAN, IAA-Leu, and ICA were detected only in LN15, while IA was detected only in LN0 (Table 1).

In leaves, N0 increased the tZOG, 2MeScZR, and iP7G concentrations relative to N15, decreased the BAP and BAP7G concentrations, and did not significantly affect the tZR, cZROG, cZ9G, DHZROG, iP9G, mT, oTR, and pT9G concentrations. cZR, DHZR, DHZ7G, K, and mTR were detected only in LN0, while iP, iPR, and 2MeSiP were detected only in LN15 (Table 1).

In leaves, GA_1_, GA_3_, GA_9_, and GA_53_ were detected in LN0. N0 increased the OPDA concentration relative to N15, decreased the H2JA and JA-Val concentrations, and did not significantly alter the JA, MEJA, OPC-6, OPC-4, JA-ILE, and JA-Phe concentrations (Table 1).

In leaves, N0 decreased the ABA concentration relative to N15, increased the ACC concentration, and did not significantly affect the ABA-GE, SA, SAG, 5DS, and ST concentrations (Table 1).

We detected 69 HRMs in RN0 and/or RN15, including 2 ABAs, 20 AUXs, 27 CKs, ACC, 7 GAs, 8 JAs, 2 SAs, and 2 SLs. Among these 27 CKs, 15 belong to isoprenoid CKs and 12 belong to aromatic CKs. A total of 4 and 14 HRMs were detected only in RN0 and RN15, respectively. Fifty-one HRMs were detected simultaneously in RN0 and RN15 (Table 1 and Figure 1A).

In roots, N0 increased the IAA-Gly concentration relative to N15, decreased the IPA, MEIAA, ILA, and IA concentrations, and did not significantly alter the IAA, TRP, TRA, OxIAA, IBA, IAA-Asp, IAA-Glu, IAA-Val, ICAld, and ICA concentrations. IAA-Val-Me was detected only in RN0, while IAN, IAM, IAA-Ala, and IAA-Leu were detected only in RN15 (Table 1).

In roots, N0 increased the tZOG, BAPR, and pT9G concentrations relative to N15, decreased the tZR, cZR, iP, iPR, and 2MeSiPR concentrations, and did not significantly alter the cZ, cZROG, 2MeScZR, iP7G, K, K9G, mT, and oT9G concentrations. KR, oT, and oTR were detected only in RN0, while tZ, 2MeScZ, DHZROG, iP9G, 2MeSiP, BAP9G, mT9G, and pT were detected only in RN15 (Table 1).

In roots, N0 increased the GA_15_ and GA_24_ concentrations relative to N15, and had no significant impacts on the GA_1_, GA_4_, and GA_19_ concentrations. GA_53_ and GA_3_ were detected only in RN15. N0 decreased the JA, OPDA, H2JA, and JA-Val concentrations relative to N15, and did not significantly alter the MEJA, OPC-6, OPC-4, and JA-ILE concentrations (Table 1).

In roots, N0 decreased the ABA and SAG concentrations relative to N15, and increased the 5DS and ACC concentrations, and did not significantly affect the SA and ST concentrations. ABA-GE was detected only in N0 (Table 1).

We obtained 16 upregulated and 16 downregulated HRMs in LN0 vs. LN15 and 13 upregulated and 29 downregulated HRMs in RN0 vs. RN15. There were 16 common differentially abundant HRMs (DAHs) between the two, among which four DAHs (IA, ILA, cZR, and OPDA) exhibited the opposite trends in changes between roots and leaves (Table 1 and Figure 1B–D).

### 2.2. Principal Coordinate Analysis (PCoA) of HRMs in Leaves and Roots

**Figure 2:**
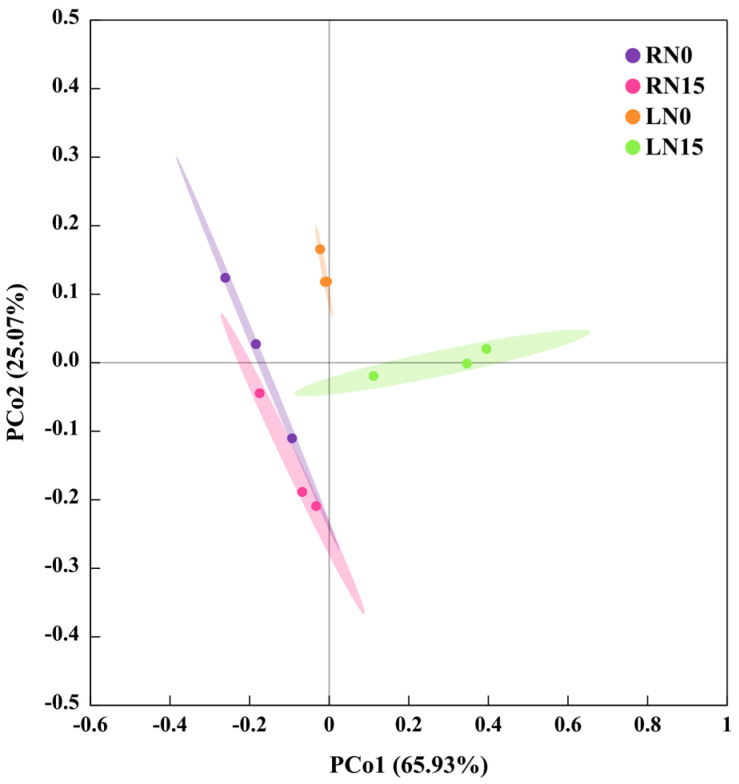
A PCoA plot of HRMs detected in leaves and roots from N0- and N15-treated *Citrus sinensis* seedlings.

To reveal the response patterns of HRMs in leaves and roots to N deficiency, a PCoA was conducted using the 80 HRMs detected in RN0, RN15, LN0, and/or LN15 (Table 1). The PCoA indicated that the four treatments, namely RN0, RN15, LN0, and L10 were separated, while the three replications per treatment were clustered. It was found that LN0 and LN15 (RN0 and RN15) were clustered on the right (left) side, suggesting that the responses of leaf and root HRMs to N deficiency were different. PCo2, which contributed 25.07% of the total variation, could separate N0 from N15 in leaves but not in roots (Figure 2).

### 2.3. Differentially Transcribed Genes (DTGs) in Leaves and Roots

As shown in Table S2, we identified 23 (40) upregulated and 10 (36) downregulated genes related to hormone metabolism in LN0 vs. LN15 (RN0 vs. RN15), including 5 (4) upregulated and 2 (10) downregulated genes involved in the AUX biosynthetic process (GO:0009851), 6 (9) upregulated and 2 (11) downregulated genes involved in the AUX metabolic process (GO:0009850), 1 (2) upregulated gene involved in the CK biosynthetic process (GO:0009691), 1 (5) upregulated and 2 (0) downregulated genes involved in the CK metabolic process (GO:0009690), 2 (9) upregulated and 1 (2) downregulated gene involved in the GA biosynthetic process (GO:0009686), 2 (9) upregulated and 1 (2) downregulated gene involved in the GA metabolic process (GO:0009685), 2 (2) upregulated and 0 (6) downregulated genes involved in the JA biosynthetic process (GO:0009695), 2 (2) upregulated and 0 (6) downregulated genes involved in the JA metabolic process (GO:0009694), 3 (0) upregulated and 0 (3) downregulated genes involved in the ETH biosynthetic process (GO:0009693), 3 (0) upregulated and 0 (3) downregulated genes involved in the ETH metabolic process (GO:0009692), 0 (6) upregulated and 3 (2) downregulated genes involved in the ABA biosynthetic process (GO:0009688), 0 (8) upregulated and 3 (3) downregulated genes involved in the ABA metabolic process (GO:0009687), 0 (1) downregulated genes involved in the SA biosynthetic process (GO:0009697), 3 (3) upregulated and 1 (3) downregulated gene involved in the SA catabolic process (GO: 0046244), 9 (5) upregulated and 2 (10) downregulated genes involved in the SA metabolic process (GO:0009696), and 0 (4) upregulated and 0 (2) downregulated genes involved in SL biosynthesis and degradation (Table S2).

## 3. Discussion

### 3.1. The Responses of AUXs in Leaves and Roots to N Deficiency

**Figure 3:**
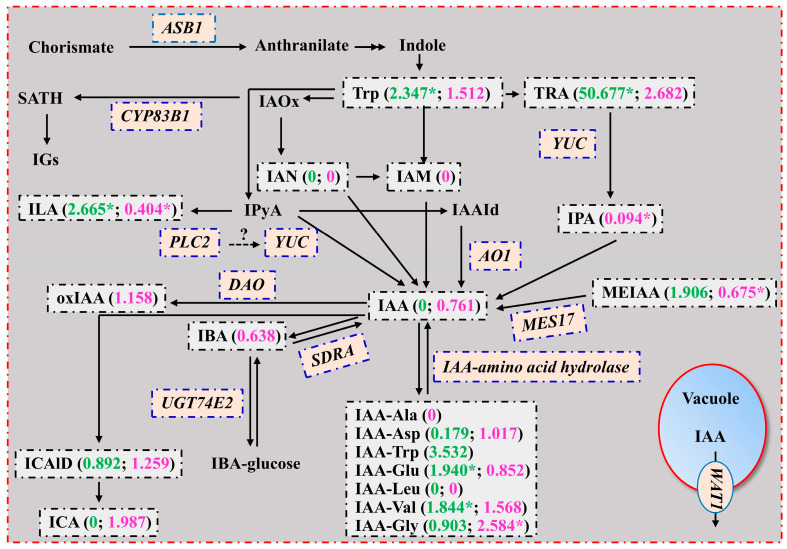
A diagram displaying the mean relative changes of AUXs in leaves (LN0/LN15; green) and roots (RN0/RN15; magenta). Data from Table 1. In this Figure, we used italics for enzymes (proteins) and plain format for HRMs (AUXs). An asterisk indicated a significant difference between LN0 (RN0) and LN15 (RN15) at *p* < 0.05. Also, an HRM was considered downregulated or upregulated when it was detected only in RN0 or LN0 (Inf) and RN15 or LN15 (fold change = 0) for a comparative group. AO1, aldehyde oxidase 1; ASB1, anthranilate synthase beta subunit 1; CYP83B1, cytochrome P450 83B1; DAO, dioxygenase for AUX oxidation; IAAId, indole-3-acetaldehyde; IAOx, indole-3-acetaldoxime; IGs, indole glucosinolates; IPyA, indole-3-pyruvic acid; MES17, methylesterase 17; oxIAA, 2-oxindole-3-acetic acid; PLC2, phosphoinositide phospholipase C 2; SATH, S-alkylthiohydroximate; SDRA, short-chain dehydrogenase/reductase; UGT74E2, UDP-glycosyltransferase 74E2; WAT1, Protein WALLS ARE THIN 1; YUC, indole-3-pyruvate monooxygenase YUCCA (refer to Ding et al. [44]; Mano and Nemoto [39]; Tognetti et al. [45]; and Woodward and Bartel [40]). The same notation will be used in Figure 4, Figure 5, Figure 6 and Figure 7.

As shown in Figure 3, four Trp-dependent IAA biosynthesis pathways have been proposed in plants, including (1) the indole-3-pyruvic acid (IPyA) pathway [Trp → IPyA → IAA or Trp → IPyA → indole-3-acetaldehyde (IAAld) → IAA], (2) the indole-3-acetaldoxime (IAO_X_) pathway, (3) the indole-3-acetamide (IAM) pathway, and (4) the tryptamine (TRA) pathway [39,40]. The current study obtained more upregulated (5) than downregulated (2) genes involved in the AUX biosynthetic process in LN0 vs. LN15 (Table S2). This does not necessarily imply that IAA biosynthesis was upregulated in LN0 vs. LN15. Further analysis suggested that the last three IAA biosynthesis pathways might be downregulated in both LN0 and RN0 because the precursors IAN and IAM in LN0 and RN0, and IPA in LN0, were not detected, and IPA in RN0 vs. RN15 was downregulated (Table 1). In the Trp → IPyA → IAA pathway, the precursor Trp is first converted to IPyA by Trp aminotransferase and IPyA is then converted to IAA by flavin monooxygenase YUCCA (YUC) [39]. Phospholipase C (PLC) plays a key role in mediating signal transduction pathways. *Arabidopsis plc2* mutants displayed enhanced expression of the AUX biosynthetic *YUCCA* genes and levels of IAA in inflorescences [41]. Interestingly, *phosphoinositide phospholipase C 2* (*PLC2*; Cs7g14760) and *indole-3-pyruvate monooxygenase YUCCA2* (*YUC2*; Cs1g23870) were upregulated in LN0 vs. LN15, and *PLC2* (Cs8g20260), *probable indole-3-pyruvate monooxygenase YUCCA8* (*YUC8*; Cs5g34410), and *probable indole-3-pyruvate monooxygenase YUCCA10* (*YUC10*; Cs5g32440) were downregulated in RN0 vs. RN15 (Table S2). Zhao [42] suggested that the Trp → IPyA → IAA pathway was the main contributor to free IAA. The upregulated ILA in LN0 implied that more IPyA was converted to ILA, thus lowering the availability of IPyA for the IAA formation catalyzed by YUC (Figure 3). Therefore, the IAA biosynthesis via the IPyA pathway might not be upregulated in LN0. In the Trp → IPyA → IAAld→ IAA pathway, the IAAld that is produced from IPyA is oxidized to IAA by aldehyde oxidase (AO). Here, we obtained 2 upregulated *aldehyde oxidase 1* (*AO1*) genes (Cs8g13760 and Cs8g13770). Seo et al. [43] found that an AUX-overproducing *superroot1* (*sur1*) mutant of *Arabidopsis thaliana* with a higher IAA concentration had a higher AO activity, and AO1 played a role in IAA biosynthesis. The downregulated ILA in RN0 vs. RN15 suggested that less IPyA was converted to ILA, thus increasing the availability of IPyA for the IAA formation catalyzed by AO (Figure 3). Therefore, the IAA biosynthesis via the IPyA pathway might be upregulated in RN0.

It was found that *Arabidopsis cyp83b1* mutants displayed enhanced levels of IAA in leaves and roots [46]. Here, we identified 1 downregulated (Cs3g07330) and 2 upregulated (Cs3g25780 and orange1.1t02796) *CYP83B1* genes in LN0 vs. LN15 and 3 downregulated (orange1.1t02083, orange1.1t02084, and orange1.1t02796) and 2 (Cs5g25880 and Cs5g25920) upregulated *CYP83B1* genes in RN0 vs. RN15 (Table S2). This might contribute to the decreased IAA level in LN0 vs. LN15 but unaltered IAA level in RN0 vs. RN15.

Indole-3-acetic acid-amino acid hydrolase IAA-LEUCINE RESISTANT1 (ILR1), ILR1-like2 (ILL2), and IAA-ALANINE RESISTANT3 (IAR3) can release IAA from IAA conjugates [47]. Here, 10 IAA–amino acid conjugates were detected from leaves and/or roots, including IAA-Ala, IAA-Leu, IAA-Asp, IAA-Gly, IAA-Glu, IAA-Val, IAA-Trp, IAA-Val-Me, IAA-Phe-Me, and IAA-Leu-Me (Table 1). Further analysis indicated that IAA-Ala and IAA-Leu (IAA-Leu) were detected in RN15 (LN15) but not in RN0 (LN0), and N0 did not affect the concentrations of the other 8 IAA-amino acid conjugates in leaves and roots with the exception that IAA-Val-ME was detected only in RN0 but not in RN15, LN0, and LN15. This agreed with the previous findings that in *Arabidopsis*, IAA-Ala and IAA-Leu were hydrolysable and contributed to the free, bioactive IAA pool, while other conjugates might have other roles [48,49], and the result that *ILR1* (Cs6g14040) was upregulated in RN0 vs. RN15 (Table S2).

Also, we identified upregulated *methylesterase (MES) 17* (*MES17*; Cs7g29470) and *short-chain dehydrogenase/reductase SDRA* (Cs2g02410) in RN0 vs. RN15 (Table S2). MES hydrolyzes MEIAA to IAA [50]. SDRA is involved in the β-oxidation of IBA to form IAA [51]. N deprivation promoted shoot-to-root IAA transport [12]. Taken together, the N deficiency-induced reduction of IAA levels in leaves might be due to elevated leaf-to-root IAA transport, increased formation of the IAA analog (ILE), and reduced biosynthesis caused by reduced precursors (IAN), while the unchanged IAA level in RN0 might be due to increased leaf-to-root IAA transport, β-oxidation of IBA, biosynthesis of IAA via hydrolysis of IAA conjugates (IAA-Leu and IAA-Ala) and precursor/storage (MEIAA), oxidation of IAAld, and reduced formation of the IAA analog (ILE) (Figure 3).

Tian et al. [11] reported that low nitrate increased IAA levels in maize roots and that high nitrate-induced inhibition of root growth could be restored by the application of IAA. Sun et al. [12] demonstrated that N deprivation improved shoot-to-root AUX transport, thus enhancing root elongation in maize seedlings. Rampey et al. [49] observed that both IAA-Leu and IAA-Ala could inhibit the root elongation of *A. thaliana* seedlings. These results indicated that N deficiency increased leaf-to-root IAA transport and maintained IAA homeostasis in roots, and increased the hydrolysis of IAA-Leu and IAA-Ala and decreased their levels in roots, thus increasing R/S [37].

### 3.2. The Responses of CKs in Leaves and Roots to N Deficiency

**Figure 4:**
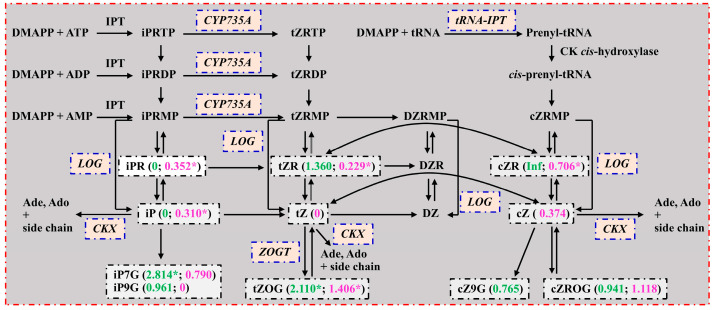
A diagram displaying the mean relative changes of CKs in leaves (LN0/LN15; green) and roots (RN0/RN15; magenta). Data from Table 1. An asterisk indicated a significant difference between LN0 (RN0) and LN15 (RN15) at *p* < 0.05. Ade, adenine; Ado, adenosine; CKX, CK oxidase/dehydrogenase; CYP735A, cytochrome P450 monooxygenase, family 735, subfamily A (CK hydroxylase); DMAPP, dimethylallyl diphosphate; DZ, dihydrozeatin; DZR, DZ riboside; DZRMP, DZ riboside 5′-monophosphate; iPRDP, iP riboside 5′-diphosphate; iPRMP, iP riboside 5′-monophosphate; iPRTP, iP riboside 5′-triphosphate; tRNA-IPT, tRNA isopentenyltransferase; LOG, CK riboside 5′-monophosphate phosphoribohydrolase; tZRDP, tZR 5′-diphosphate; tZRMP, tZR 5′-monophosphate; tZRTP, tZR 5′-triphosphate; ZOGT, zeatin O-glucosyltransferase (refer to Hirose et al. [53] and Sakakibara [38]).

Free CK bases are biologically active in plants, including tZ, cZ, iP, dihydrozeatin (DZ), BAP, K, mT, oT, and pT, but once they are ribosylated, they are converted to transport forms with weaker CK activities [38,52]. As shown in Table 1 and Figure 4, we obtained downregulated iP and BAP in LN0 vs. LN15, and K was detected only in LN0; downregulated iP in RN0 vs. RN15 and tZ and pT (oT) were detected only in RN15 (RN0). Generally viewed, N0 decreased the accumulation of free CK bases in leaves and roots relative to N15. To become bioactive, CK nucleotides are converted to nucleobase forms by dephosphorylation and deribosylation or are directly catalyzed to the bioactive forms by the CK riboside 5′-monophosphate phosphoribohydrolase called LOG [38,53]. CK dehydrogenase (CKX) is involved in the degradation of CKs by cleavage of the side chain. tRNA isopentenyltransferase (tRNA-IPT) is involved in cZ biosynthesis. CK hydroxylase catalyzes the conversion of iP nucleotides to tZ nucleotides. Zeatin O-glucosyltransferase (ZOGT) catalyzes the conversion of CKs from bioactive to inactive forms [38]. We obtained 1 upregulated *LOG1* (Cs3g25930) involved in the CK biosynthetic process and 1 upregulated (*LOG1*) and 2 downregulated [*CK dehydrogenase 7* (*CKX7*; Cs4g06150) and *CKX1* (orange1.1t00627)] genes involved in the CK metabolic process in LN0 vs. LN15, as well as 2 upregulated genes [*tRNA dimethylallyltransferase 2* (*IPT2*; Cs3g07650) and *CK hydroxylase* (*CYP735A1*; Cs9g17460)] involved in the CK biosynthetic process and 5 upregulated genes [*IPT2*, *CYP735A1*, *CKX3* (*Cs4g14450*), and 2 *ZOGT* (Cs4g16640 and orange1.1t02338)] involved in the CK metabolic process in RN0 vs. RN15 (Table S2). The downregulation of iP in LN0 vs. LN15 might be caused by the decreased biosynthesis via the dephosphorylation and deribosylation of iPRMP, as indicated by the downregulation of iPR, and the increased formation of iP-conjugates (iP7G) (Figure 4). The downregulation of iP (tZ) in RN0 vs. RN15 might be caused by the decreased biosynthesis via the dephosphorylation and deribosylation of iPRMP (tZPRM), as indicated by the downregulation of iPR (tZR), and the increased degradation of iP (tZ) catalyzed by CKX, as indicated by the upregulated expression of *CKX3* (Figure 4). Also, the increased formation of tZ conjugates (tZOG) catalyzed by ZOGT might contribute to the downregulation of tZ in RN0 vs. RN15 (Figure 4). However, the elevated accumulation of tZOG in LN0 vs. LN15 could not be explained in this way. *CKX*-overexpressing transgenic tobacco and *Arabidopsis* plants had increased breakdown of CKs and reduced accumulation of CKs (iP and zeatin), exhibiting reduced shoot growth and increased root growth [13]. The vegetative characteristics of transgenic maize overexpressing a *zeatin O-glucosyltransferase* gene (*ZOG1*) from *Phaseolus lunatus* resembled those of CK-deficient plants, including decreased shoot growth, and increased root mass and branching [54]. Wang et al. [55] suggested that an N starvation-induced decrease in CK (zeatin) levels in rice roots improved seminal root growth by enhancing meristem cell proliferation and cell elongation. Tian et al. [56] reported that low nitrate reduced the level of zeatin + zeatin nucleotide in the roots of maize inbred line 478, but not in the roots of maize inbred line Wu312, that nitrate with a concentration of 5 mM or higher inhibited root elongation in 478, whereas root elongation in Wu312 was repressed only at 20 mM nitrate, and that exogenous 6-benzylaminopurine (6-BA) completely counteracted the stimulating action of low nitrate on root elongation. Gan and Amasino [57] showed that overexpressing *IPT* in *A. thaliana* inhibited leaf senescence by increasing CK (iP) levels. These results suggested that N0 decreased the levels of CKs in leaves (iP and BAP) and roots (iP, tZ, and pT), thus inhibiting leaf growth, accelerating leaf senescence, and increasing R/S [37].

### 3.3. The Responses of GAs in Leaves and Roots to N Deficiency

**Figure 5:**
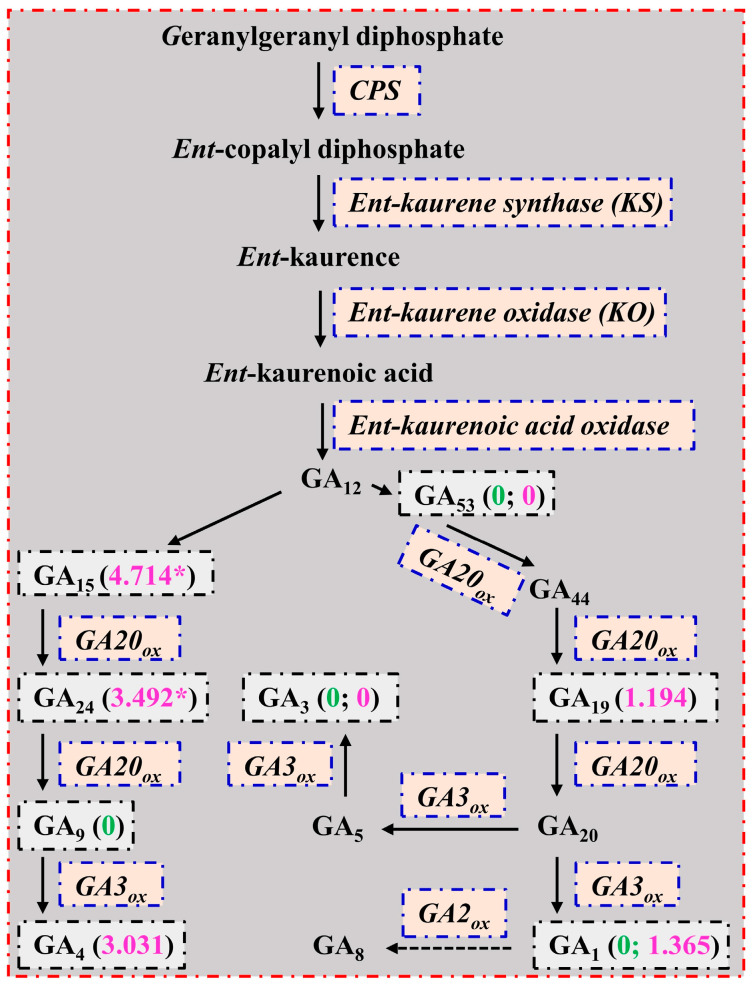
A diagram displaying the mean relative changes of GAs in leaves (LN0/LN15; green) and roots (RN0/RN15; magenta). Data from Table 1. An asterisk indicated a significant difference between LN0 (RN0) and LN15 (RN15) at *p* < 0.05. CPS, *ent*-copalyl diphosphate synthase; GA2_ox_, GA 2-oxidase; GA3_ox_, GA 3-oxidase; GA20_ox_, GA 20-oxidase (refer to Binenbaum et al. [58] and Yamaguchi [59]).

Gibberellin A_1_, GA_3_, GA_4_, and GA_7_ are considered to be bioactive in plants [58,59]. Qi et al. [60] demonstrated that a rice *APETALA2 (AP2)/ETH-Responsive Element Binding Factor (ERF)* gene (*OsEATB*) could restrict internode elongation by lowering GA (GA_1_, GA_4_, GA_9_, GA_12_, GA_19_, GA_20_, GA_24_, and GA_53_) biosynthesis and accumulation due to downregulated expression of a rice *ent-copalyl diphosphate synthase* (*CPS*) gene (*OsCPS*). Swain et al. [61] reported that the GA level was not altered in transgenic *Arabidopsis* plants with increased *ent-kaurene oxidase* (*KO*) expression. We detected 1 downregulated *CPS, chloroplastic* (Cs5g15530) and 2 upregulated *KO*, *chloroplastic* (*KO1*; orange1.1t01909 and orange1.1t01910) genes in LN0 vs. LN15 (Table S2). These findings suggested that N deficiency lowered the expression of *CPS*, thus lowering GA (GA_1_, GA_3_, GA_9_, and GA_53_) biosynthesis and levels in leaves (Table 1 and Figure 5), and hence inhibiting leaf (shoot) growth [37]. This agreed with the work that low N lowered growth and GA (GA_4_, GA_12_, GA_15_, GA_24_, and GA_34_) biosynthesis and accumulation in *Arabidopsis* seedlings [1].

Unlike in leaves, we detected 9 upregulated and 2 downregulated genes involved in the GA biosynthetic process in RN0 vs. RN15 (Table S2). Achard et al. [62] found that the *Arabidopsis serine/threonine-protein kinase ctr1–1* mutant contained decreased concentrations of bioactive GAs (GA_1_ and GA_4_) and increased concentrations of some intermediate GAs (GA_24_ and GA_53_) accompanied by smaller vegetative rosette sizes and shorter petiole lengths. Overexpression of *AtCPS* and *AtKS* in *Arabidopsis* led to an increase in *ent* kaurene production but did not result in an increase in bioactive GA production [63]. Overexpression of *GA20_ox_* conferred a GA-overproduction phenotype in *Arabidopsis* accompanied by increased concentrations of GA_1_, GA_9_, and GA_20_ and unaltered concentrations of GA_4_ [64]. Radi et al. [65] found that overexpression of a pumpkin *GA3_ox1_* in *Arabidopsis* led to increased GA_4_ concentrations. Overexpression of a pea *GA3_ox1_* in tobacco plants resulted in an increase in GA_1_ levels [66]. Vidal et al. [67] indicated that overexpression of a citrus *GA20_ox1_* in tobacco enhanced the biosynthesis of GAs through the non-13-hydroxylation pathway but decreased the biosynthesis through the early-13-hydroxylation pathway, thus causing GA_4_ to become the main bioactive GA in the transgenic tobacco plants. We found that the GA_4_ and GA_1_ concentrations displayed an upward trend in RN0 vs. RN15, while the GA_3_ level was significantly decreased in RN0 vs. RN15 and the level of GA_1_ (GA_4_) in RN0 was much higher than that of GA_3_. These results suggested that the upregulation of *CTR1* (Cs4g05990), *CPS*, *chloroplastic* (Cs5g31210), *KS* (orange1.1t03278), *KO1* (orange1.1t01909 and orange1.1t01910), *GA20_ox1_* (Cs9g16520), *GA20_ox2_* (orange1.1t00272), and *GA3_ox1_* (Cs4g20350) in RN0 vs. RN15 might lead to a redirection of GA biosynthesis to GA_1_ and GA_4_, thereby preventing their decline and maintaining them at higher levels. Lv et al. [20] reported that N limitation increased GA_3_ concentrations in wheat roots. However, Wang et al. [19] reported that N deprivation reduced the levels of GA_1_, GA_3_, and GA_4_ in maize roots. GA deficiency can reduce crop NUE [68]. Therefore, the relatively stable GA levels in RN0 vs. RN15 might be beneficial for improving R/S, NUE, and the ability to acquire N. Evidence shows that IAA can promote GA biosynthesis and accumulation [69]. The different responses of leaf and root GAs to N deficiency agreed with our results that LN0 reduced IAA concentration in leaves but did not significantly alter its concentration in roots (Table 1).

### 3.4. The Responses of JAs in Leaves and Roots to N Deficiency

**Figure 6:**
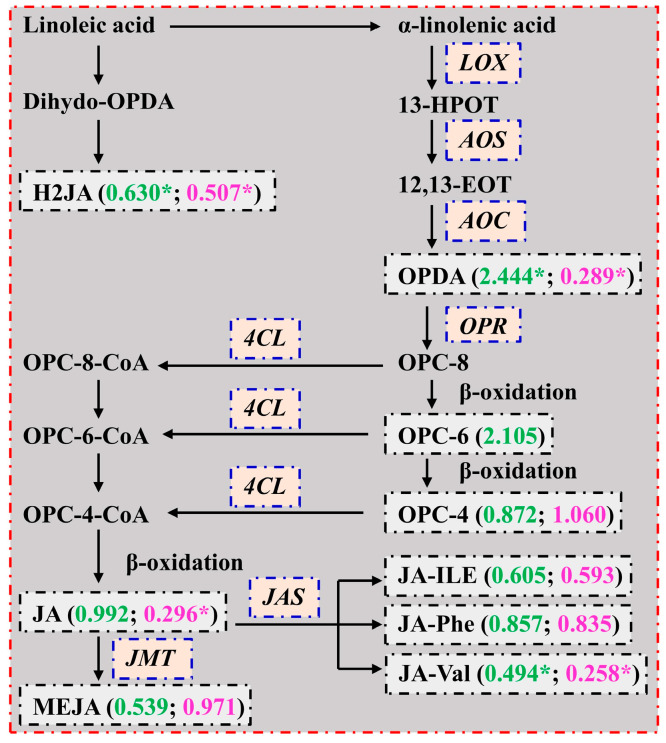
A diagram displaying the mean relative changes of JAs in leaves (LN0/LN15; green) and roots (RN0/RN15; magenta). Data from Table 1. An asterisk indicated a significant difference between LN0 (RN0) and LN15 (RN15) at *p* < 0.05. AOC, allene oxide cyclase; AOS, allene oxide synthase; 4CL, 4-coumarate--CoA ligase; Dihydro-OPDA, dihydro-12-oxo-phytodienoic acid; 12,13-EOT, 12,13-epoxyoctadecatrienoic acid; 13-HPOT, (13S)-hydroperoxyoctadecatrienoic acid; JAS, jasmonoyl amino acid synthetase; JMT, jasmonate O-methyltransferase; LOX, lipoxygenase; OPDA, 12-oxo-phytodienoic acid; OPR, 12-oxophytodienoate reductase (refer to Kienow et al. [73]).

We detected two upregulated (*4-coumarate--CoA ligase-like 5* (*4CLL5*; Cs7g21790) and *4CLL9* (Cs5g06990)) and six downregulated (four *allene oxide cyclase* (*AOC*), *chloroplastic* (Cs3g06080, Cs6g18900, Cs6g18910, and novel.2517), *lipoxygenasem (LOX) 2, chloroplastic* (*LOX2*; novel.2136), and *Protein GRIM REAPER* (*GRI*; novel.1617)) genes involved in the JA biosynthetic process in RN0 vs. RN15, and upregulated *4CLL5* (Cs3g27010) and *4CLL9* (Cs5g06980) in LN0 vs. LN15 (Table S2). Transgenic tomato plants silencing *loxD* had lower LOX activity and JA concentration [70]. Hazman et al. [71] reported that two *aoc* rice mutants (*hebiba* and *cpm2*) had decreased levels of JA, OPDA, and JA-ILE than those of their wild-type plants under control conditions and 6 h of salinity stress with the exception that the OPDA level did not differ between the two under the control conditions. Silencing of *Gh4CL30* in cotton inhibited JA biosynthesis in the uninfected plants but not in the *Verticillium dahlia*-infected plants [72]. Therefore, JA biosynthesis might be downregulated in RN0 vs. RN15. This was supported by our results that RN0 had lower concentrations of JA, OPDA, H2JA, and JA-Val than RN15 (Table 1 and Figure 6). Although the expression levels of *4CLL5* and *4CLL9* were upregulated in LN0 vs. LN15, N0 only increased the concentration of OPDA and decreased or did not alter the concentrations of the other JAs. This suggested that the biosynthesis of JAs was not upregulated in LN0 vs. LN15, but it might be redirected to OPDA biosynthesis, and that the *4CLL5* and *4CLL9* were not the rate limiting enzymes for the biosynthesis of JAs in leaves. It is known that JA can inhibit plant growth [15]. The downregulated JA in RN0 vs. RN15 and the unaltered JA in LN0 vs. LN15 suggested that N deficiency promoted root growth (relative to shoot growth), thus increasing R/S [37].

The current finding that LN0 had increased OPDA levels and unaltered JA and JA-ILE levels relative to LN15 agreed with the findings that drought increased the accumulation of OPDA in *Arabidopsis* seedlings rather than JA [74], and that salt-stress increased OPDA levels but did not affect JA and JA-ILE levels in rice shoots [71]. A study indicated that OPDA negatively regulated *Arabidopsis* stomatal opening independently and synergistically with ABA, and that the *opr3* mutant lines with increased accumulation of OPDA displayed reduced stomatal aperture and enhanced drought tolerance [74]. Thus, the increased accumulation of OPDA in LN0 might cause stomatal closure [37], thus lowering transpiration water loss and enhancing N deficiency tolerance. Zhang and Turner [75] reported that exogenous OPDA reduced the leaf area of the wild-type *Arabidopsis* plants, but not of the *opr3* plants, concluding that the OPDA-induced reduction in the leaf area of the wild-type plants might be due to its conversion to JA or one of its metabolites. However, Mueller et al. [76] reported that OPDA reduced the overall growth of wild-type *Arabidopsis* seedlings, particularly root growth. Root growth inhibition by OPDA was less in the *opr3* than in the wild-type. Wu et al. [77] indicated that in rice seedlings, exogenous applications of MeJA lowered the uptake of N by the roots and the root-to-shoot translocation of recently-absorbed ^15^N.

To conclude, N deficiency reduced the biosynthesis and accumulation of JAs, while it might redirect to the OPDA biosynthesis. The different operations of the JA biosynthesis pathway in N-deficient leaves and roots might confer citrus N deficiency tolerance by improving R/S, NUE, N distribution in roots, and the ability to acquire N [2,37].

### 3.5. The Responses of ETH in Leaves and Roots to N Deficiency

It was reported that the overexpression of the citrus *ACC synthase* (*ACS*) gene (*CiACS4*) led to a dwarfing phenotype, increased ETH release, and decreased IAA, GA_3_, and GA_20_ concentrations in transgenic lemon (*Citrus limon*) plants, while the inhibition of *CiACS4* expression resulted in exactly the opposite [78]. Wi and Park [79] reported that the antisense expression of carnation *ACS* or *ACC oxidase* (*ACO*) attenuated stress-induced senescence by lowering ETH formation in transgenic tobacco plants. Therefore, N0 might reduce ETH biosynthesis in roots, as indicated by the downregulated expression of *ACS8* (Cs3g16400), *ACO* (Cs1g15700), and *ACO5* (Cs3g20140; Table S2). As well as this, the upregulation of *CTR1* (a negative regulator of ETH response) in RN0 vs. RN15 (Table S2) might contribute to the lower ETH formation. This agreed with our results that N0 increased or did not affect the concentrations of 5 out of 7 GAs in roots (Table 1), and with the reports that ETH formation was lowered in N-starved maize roots [8]. Tian et al. [36] reported that in *Arabidopsis*, the high N-induced downregulation of the *high affinity nitrate transporter NRT2.1* and the upregulation of the *low affinity nitrate transporter NRT1.1* in roots could be inhibited by the ETH synthesis antagonist, aminoethoxyvinylglycine. In *Arabidopsis* roots, *NRT2.1* expression was upregulated by aminoethoxyvinylglycine and N starvation and downregulated by ETH [80]. Zhang et al. [81] indicated that the application of ethephon reduced maize N uptake. Léran et al. [82] showed that *Arabidopsis* NRT1.1 was a bidirectional transporter and was involved in root-to-shoot nitrate transport. Recently, it was found in our laboratory that N0 upregulated the expression of *NRT2.4* (Cs8g16010 and orange1.1t02415) and *NRT2.5* (Cs7g09040) and downregulated the expression of *NRT1.1* (Cs5g09050) in roots [6]. These results suggested that N0 decreased ETH biosynthesis, thereby delaying root senescence and improving the R/S, the capacity of roots to acquire N and the distribution of N in roots [37]. The increased accumulation of ACC in RN0 vs. RN15 (Table 1) might be mainly caused by its decreased utilization for ETH biosynthesis, as indicated by the downregulated expression of *ACO* and *ACO5* and the upregulated expression of *CTR1*. This suggested that *ACS8* was not the rate limiting enzyme for ACC biosynthesis in RN0.

Rauf et al. [83] found that in *A. thaliana*, waterlogging triggered the *NAC transcription factor Speedy Hyponastic Growth* (*SHYG*), which then activated the expression of *ACO5* and *ASC*, thus increasing ETH biosynthesis. Here, N0 upregulated the expression of *ACS2* (orange1.1t00414), *ACO* (Cs2g20590), and *SHYG* (Cs5g26130) and increased the levels of ACC in leaves (Table S2), thereby enhancing ETH formation and release. Varsani et al. [84] indicated that OPDA enhanced the expression of ETH biosynthesis genes (*ACS2*, *ACS6*, and *ACO15*) in maize plants. Thus, the upregulation of OPDA in LN0 vs. LN15 might contribute to the increased ETH biosynthesis. The increase in ETH biosynthesis in LN0 agreed with our results that N0 decreased the levels of GA_1_, GA_3_, GA_9_, and GA_53_ in leaves (Table 1), and with the reports that N deficiency increased ACS activity and ETH evolution in mustard leaves [32], and N deficiency accelerated *C. sinensis* leaf senescence [25]. However, Iqbal et al. [32] reported that the application of ethephon increased leaf stomatal conductance, photosynthesis, area, and plant growth in mustard. Thus, the increase in ETH formation in LN0 might be an adaptive response to N deficiency.

### 3.6. The Responses of ABAs in Leaves and Roots to N Deficiency

**Figure 7:**
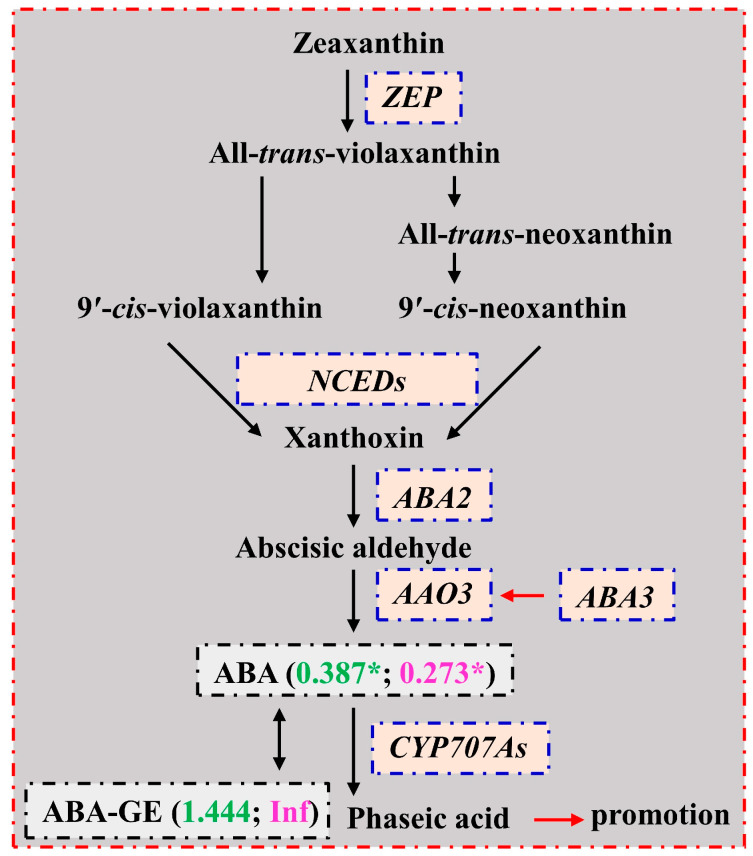
A diagram displaying the mean relative changes of ABAs in leaves (LN0/LN15; green) and roots (RN0/RN15; magenta). Data from Table 1. An asterisk indicated a significant difference between LN0 (RN0) and LN15 (RN15) at *p* < 0.05. AAO3, abscisic-aldehyde oxidase; ABA2, xanthoxin dehydrogenase; ABA3, molybdenum cofactor sulfurase; CYP707A, ABA 8′-hydroxylase; NCED, 9-cis-epoxycarotenoid dioxygenase; ZEP, zeaxanthin epoxidase (refer to Chen et al. [91] and Watanabe et al. [92]).

We identified 2 downregulated (*zeaxanthin epoxidase* (*ZEP*), *chloroplastic* (Cs1g22620) and *NDR1/HIN1-like protein 6* (*NHL6*; novel.774)) and 6 upregulated (3 *ZEP*, *chloroplastic* (orange1.1t04051, orange1.1t05125 and orange1.1t04849), 2 *AO1* (Cs8g13760 and Cs8g13770), and *molybdenum cofactor sulfurase* (*ABA3*; Cs4g12520)) genes involved in the ABA biosynthetic process, as well as 1 downregulated (Cs3g23530) and 2 upregulated (Cs3g21210 and Cs1g24480) *ABA 8′-hydroxylases 3* (*CYP707A3*) genes involved in the oxidative degradation of ABA in RN0 vs. RN15 (Table S2). Transgenic *Nicotiana plumbaginifolia* plants overexpressing a *ZEP* and expressing an antisense *ZEP* displayed elevated and decreased ABA concentrations in transgenic seeds, respectively [85]. The final step in ABA biosynthesis is the oxidation of abscisic aldehyde to ABA. This reaction is catalyzed by abscisic aldehyde oxidase 3 (AAO3), which requires a molybdenum cofactor that is biosynthesized by the ABA3 [86]. This reaction can also be catalyzed by AO [87]. Overexpression of *NHL6* increased the levels of ABA in transgenic *Arabidopsis* plants, but the levels of ABA did not differ between the *nhl6* and the wild-type plants [88]. Overexpression of *PvCYP707As* in *Nicotiana sylvestris* led to increased phaseic acid (PA) concentrations but decreased ABA concentrations in transgenic plants [89]. Therefore, the decreased ABA levels in RN0 vs. RN15 (Table 1) might be caused by the elevated degradation, rather than by the decreased biosynthesis (Figure 7). The decrease in ABA levels in RN0 might also be due to the increased formation of the inactive storage form ABA-GE by the glucosylation of ABA and the decreased release of ABA from ABA-GE, because ABA-GE was detected only in RN0 (Table 1). Lee et al. [90] reported that under low nitrate conditions, the *NITROGEN RESPONSE DEFICIENCY 1* (*nid1*) knockout *Arabidopsis* mutants displayed longer primary roots and more lateral roots than the Col-0 plants but lower accumulation of ABA. The decrease in ABA levels in RN0 might contribute to the increase in R/S during N deficiency.

Here, we identified 3 downregulated genes (2 *ZEP, chloroplastic* (Cs4g20590 and Cs4g20560) and *9-cis-epoxycarotenoid dioxygenase NCED1, chloroplastic* (Cs2g03270)) involved in the ABA biosynthetic process in LN0 vs. LN15 (Table S2). Tan et al. [93] found that the ABA levels were lower in the maize *vp14* embryos than in the wild-type embryos, and that the *vp14* phenotype could be rescued by ABA. The gene encodes NCED1. Thus, the decrease in ABA levels in LN0 vs. LN15 might be caused by a decrease in ABA biosynthesis. This agreed with the reports that ABA levels in *Catalpa bungei*, rice, and pea leaves did not significantly alter under N deficiency but displayed a decreased trend [94,95,96]. Studies indicated that ABA addition could indirectly enhance leaf growth via reducing ETH formation [97], or directly repress leaf expansion via lowering cell wall extensibility [98]. In cucumbers, Oka et al. [23] found that ABA inhibited leaf senescence under N starvation. Taken together, N deficiency caused a decrease in ABA levels and an increase in ETH biosynthesis in LN0 vs. LN15, thus reducing leaf growth and accelerating leaf senescence. However, Zakari et al. [29] indicated that the N deficiency-induced increase in ABA levels in rice leaves was the important factor for the accelerated leaf senescence under N deprivation, and that the exogenous application of ABA could accelerate rice leaf senescence.

### 3.7. The Responses of SLs in Leaves and Roots to N Deficiency

As shown in Table S2, we identified 1 downregulated (*carotenoid cleavage dioxygenase 8 (CCD8) homolog B, chloroplastic* (*CCD8b*; Cs4g19470)) and 3 upregulated (*β-carotene isomerase D27, chloroplastic* (Cs5g30540), *CCD7*, *chloroplastic* (*Cs1g25090*) and *CCD8b* (Cs4g19460)) genes involved in SL biosynthesis, and 1 upregulated *SL esterase D14* (Cs3g16030) and 1 downregulated *probable strigolactone esterase DAD2* (Cs4g16860) gene involved in SL degradation in RN0 vs. RN15. A rice *d14* mutant with an increased level of 2′-*epi*-5-deoxystrigol displayed enhanced outgrowth of tillers [99]. Therefore, the elevated level of 5DS in RN0 vs. RN15 might be caused by the elevated biosynthesis and the reduced degradation. Additionally, the ST level in RN0 displayed an increased trend, whereas the 5DS and ST levels in LN0 displayed a decreased trend (Table 1). This agreed with the results that in sorghum, N starvation increased 5DS biosynthesis and accumulation in roots and its exudation by roots but it did not alter 5DS concentration in shoots [100], and with our results that the levels of GAs were reduced in LN0 vs. LN15, but generally displayed an increased trend in RN0 vs. RN15 (Table 1), because GA can improve SL biosynthesis by inducing the expression of *D27* [101]. SLs are biosynthesized mainly by roots [21]. Sun et al. [10] demonstrated that N starvation-induced accumulation of SLs improved NUE. The different responses of leaf and root SLs to N deficiency might confer citrus N deficiency tolerance by increasing R/S, NUE, and the ability to acquire N.

### 3.8. The Responses of SAs in Leaves and Roots to N Deficiency

Xia et al. [102] showed that an *Arabidopsis aspartic proteinase CDR1* (*CDR1-D*) dominant mutant had a dwarf stature and greatly elevated levels of SA and its glucoside than the wild-type plants. *Lipase-like PAD4*, *serine/threonine-protein kinase PCRK1*, and *Protein SAR DEFICIENT 1* (*SARD1*) can upregulate *isochorismate synthase 1* (*ICS1*), one key gene involved in SA biosynthesis [103]. *Arabidopsis* plants overexpressing both *PAD4* and *ENHANCED DISEASE SUSCEPTIBILITY 1* (*EDS1*) were stunted compared to wild-type plants accompanied by increased accumulation of free SA [104]. Vogelmann et al. [105] observed early senescence and cell death, as well as increased concentrations of SA + SA glucoside (total SAs) in *Arabidopsis senescence-associated ubiquitin ligase1* (*saul1*) mutants but not in *saul1-1/pad4 double*. They suggested that the PAD4-dependent SA pathway was necessary for *saul1* senescence and cell death. The upregulation of *PAD4* (Cs1g08280), *PCRK1* (Cs6g12050), *SARD1* (Cs7g27120), and *CDR1* (Cs5g18300) in LN0 vs. LN15 (Table S2) implied that the biosynthesis of SA might be upregulated in LN0. As shown in Table 1 and Table S2, we obtained 3 upregulated (2 *Protein DMR6-LIKE OXYGENASE 2* (*DLO2*; Cs9g14480 and orange1.1t01963) and 1 *Protein DOWNY MILDEW RESISTANCE 6* (*DMR6;* Cs5g16310)) and 1 downregulated (*DLO1*; Cs5g28730) gene involved in the SA catabolic process, 2 upregulated *UDP-glycosyltransferase 74F2* (*UGT74F2*; Cs5g21200 and Cs5g21220) genes involved in the conversion (glucosylation) of SA to SAG and SA glucose ester (SGE) [106], and an unchanged SA level and an increasing trend in SAG level in LN0 vs. LN15. Zhang et al. [107] found that *Arabidopsis salicylate 3-hydroxylase DLO1* (*s3h*) knockout mutants failed to convert SA to 2,3-dihydroxybenzoic acid (2,3-DHBA) sugar conjugates, contained high concentrations of SA and its sugar conjugates, and displayed accelerated leaf senescence, while the reverse was the case for the gain-of-function lines. *Arabidopsis* plants overexpressing *UGT74F2* showed decreased accumulation of free SA and elevated susceptibility to *Pseudomonas syringae* [108]. The upregulation of *UGT74F2* (Cs5g21200 and Cs5g21220) and the downregulation of *DLO1* (Cs5g28730) in LN0 vs. LN15 implied that the glucosylation and catabolism of SA might be upregulated and downregulated, respectively, in LN0. These results suggested that the increase in SA glycosylation induced by N0 offsets the increase in SA biosynthesis and decrease in SA catabolism induced by N0, thereby keeping the SA concentration in LN0 unchanged, and that the increased SA biosynthesis and glucosylation, reduced catabolism, and an increased trend in the level of SA + SAG (Table 1) might be responsible for the reduced growth and accelerated senescence in these leaves. Unlike the leaves, the biosynthesis of SA might be downregulated in RN0 vs. RN15, as indicated by the downregulated *GRI* involved in the SA biosynthetic process, as well as the 2 downregulated *CDR1* (Cs5g18300 and Cs5g18330) genes (Table S2). Also, we obtained 3 upregulated (2 *DLO1* (Cs5g28720 and Cs5g28750) and 1 *DLO2* (Cs9g14500)) and 3 downregulated (2 *DLO1* (Cs5g28710 and Cs1g12310) and 1 *DLO2* (Cs5g28780)) genes involved in the SA catabolic process, as well as 2 upregulated (Cs2g18300 and novel.306) and 3 downregulated (Cs2g18240, Cs5g21200, and Cs5g21220) *UGT74F2* genes in RN0 vs. RN15 (Table S2). These results suggested that the biosynthesis and glucosylation of SA might be reduced in RN0 vs. RN15, thereby keeping the SA concentration in RN0 unchanged while decreasing its SAG concentration (Table 1). It is known that moderate levels of SA promote root and shoot growth, while higher levels of SA have the opposite impact [17]. Chen et al. [35] showed that low N increased the concentrations of SA in cotton main and lateral roots, that the main root average diameter (lateral root length) was negatively correlated with the SA concentration, and that moderate N levels improved root growth by lowering SA concentrations in the main and lateral roots. Conesa et al. [5] indicated that 50 µM of SA inhibited *Arabidopsis* root growth. Therefore, the N deficiency-induced downregulation of SA biosynthesis and glucosylation and reduction of SAG concentration in roots might be beneficial for root growth, thereby improving R/S.

## 4. Materials and Methods

### 4.1. Plant Materials and Treatments

Plant materials and treatments referred to Lai et al. [6]. Six weeks after germination of sweet orange (*Citrus sinensis* (L.) Osbeck cv. Xuegan) seeds, the uniform seedlings were transplanted into 6 L flowerpots (2 plants per flowerpot) containing sand. The seedlings were grown in an unheated greenhouse at the Fujian Agriculture and Forestry University, Fuzhou, China (26°5′ N, 119°14′ E) under a natural light environment. The annual average temperature, relative humidity, and sunshine hours were ~20 °C, 76%, and 1600 h, respectively [109]. One week after transplantation, the seedlings were supplied thrice weekly with quarter-, half-, and full-strength nutrient solutions for 1, 2, and 3 weeks, respectively, until dripping (~500 mL pot^−1^). The full-strength nutrient solution refers to the nutrient solution used for the 15 mM N treatment. Seven weeks after transplantation, they were provided thrice weekly with nutrient solutions containing 0 (N0) or 15 (N15 or control) mM N [i.e., macronutrients (Table 2) and micronutrients: 10 μM H_3_BO_3_, 20 μM Fe-EDTA, 2 μM MnCl_2_, 2 μM ZnSO_4_, 0.5 μM CuSO_4_, and 0.065 μM (NH_4_)_6_Mo_7_O_24_] until dripping. There were 10 flowerpots of seedlings per treatment arranged in a randomized design. After 10 weeks of N treatments, the recently fully expanded (~7-week-old) leaves and ~ 5 mm long white root apices were used for all determinations except for N in roots. Leaves (petioles, winged leaves, and midribs removed) and ~5 mm long white root apices were harvested at sunny noon and immediately frozen in liquid N_2_, then stored at −80 °C until extraction of hormones and RNA.

### 4.2. Extraction and Determination of HRMs in Leaves and Roots

Six frozen leaf samples (2 treatments × 3 biological replicates) and 6 frozen root samples (2 treatments × 3 biological replicates) were sent to Wuhan MetWare Biotechnology Co., Ltd. (Wuhan, China) (https://www.metware.cn/, accessed on 1 June 2023) for extraction and determination of HRMs. Equal amounts of leaves (roots) from 3 plants (1 per pot) were mixed as a biological replicate. After the frozen samples were ground to a fine powder in liquid N_2_ using a mixer mill (30 Hz, 1 min; MM400, Retsch GmbH, Haan, Germany), 50 mg of the powder was accurately weighed into a 2 mL tube containing 1 mL of methanol/water/formic acid (15:4:1, *v*:*v*:*v*) and 10 μL of internal standard mixed solution (100 ng/mL). The mixture was vortexed for 10 min. After centrifugation at 4 °C and 16,000× *g* for 5 min, the supernatant was collected, evaporated with a CentriVap centrifuge concentrator (Labconco, Kansas City, MO, USA) to dryness, redissolved in 100 μL of 80% methanol (*v*/*v*), and filtered through a 0.22 μm filter. The yielding filtrate was used for the determination of HRMs [110].

Hormones and related metabolites were detected using a UPLC-ESI-MS/MS system (UPLC, ExionLC™ AD, https://sciex.com.cn/, accessed on 1 June 2023; MS, QTRAP^®^ 6500+ System, https://sciex.com.cn/). The separations of HRMs were performed with the Waters ACQUITY UPLC HSS T3 C18 column (100 mm × 2.1 mm i.d., 1.8 µm) in a mobile phase consisting of water with 0.04% acetic acid (A) and acetonitrile with 0.04% acetic acid (B). The gradient program was as follows: started at 5% B + 95% A (0–1 min), increased to 95% B + 5% A (1–8 min), held at 95% B + 5% A (8–9 min), and finally ramped back to 5% B + 95% A (9.1–12 min). Injection volume, flow rate, and temperature were 2 μL, 0.35 mL min^−1^, and 40 °C, respectively [111].

The ESI source operation parameters were as follows: ion source, ESI+/−; source temperature, 550 °C; ion spray voltage (IS), −4500 V for negative ion mode and 5500 V for positive ion mode; and curtain gas (CUR), 35 psi. HRMs were analyzed using scheduled multiple reaction monitoring (MRM). Data acquisitions were performed using the Analyst 1.6.3 software (Sciex). The Multiquant 3.0.3 software (Sciex) was used to quantify all metabolites. Mass spectrometer parameters including the declustering potentials (DP) and collision energies (CE) for individual MRM transitions were obtained with further DP and CE optimization. A specific set of MRM transitions were monitored for each period according to the metabolites eluted within this period [112,113,114].

### 4.3. RNA-Seq and Analysis

A total of 6 frozen leaf samples (2 treatments × 3 biological replicates) and 6 frozen root samples (2 treatments × 3 biological replicates) were sent to Novogene Corporation (Beijing, China) for RNA extraction and RNA-Seq. The samples were sequenced on an Illumina (Illumina Inc., San Diego, CA, USA) Novaseq platform and 150 bp paired-end reads were generated [25]. RNA was extracted using Recalcirtant Plant Total RNA Extraction Kit (Bioteke Corporation, Beijing, China) following the manufacturer’s instructions [115]. High-quality clean reads were mapped to the *C. sinensis* v2.0 genome (http://citrus.hzau.edu.cn/download.php, accessed on 1 June 2023) using HISAT2. DESeq2 1.22.2 by default was used for differential transcriptional analysis between two groups. The yielding *p*-values were adjusted using the Benjamini and Hochberg’s method to control the false discovery rate (FDR). Genes with both FDR < 0.05 and |log_2_(fold change)| > 1 were considered differentially transcribed. The reliability of the RNA-Seq data was validated by qRT-PCR, which was run in 2 technical replicates and 3 biological replicates using *U4/U6 small nuclear ribonucleoprotein PRP31* (Cs7g08440) and *actin* (Cs1g05000) as the internal standards [6,25].

### 4.4. Statistical Analysis

Results were the means ± SE of three replicates. The unpaired *t*-test was performed for comparison between means. The PCoA was implemented by ChiPlot (https://www.chiplot.online, accessed on 1 June 2023).

## 5. Conclusions

**Figure 8:**
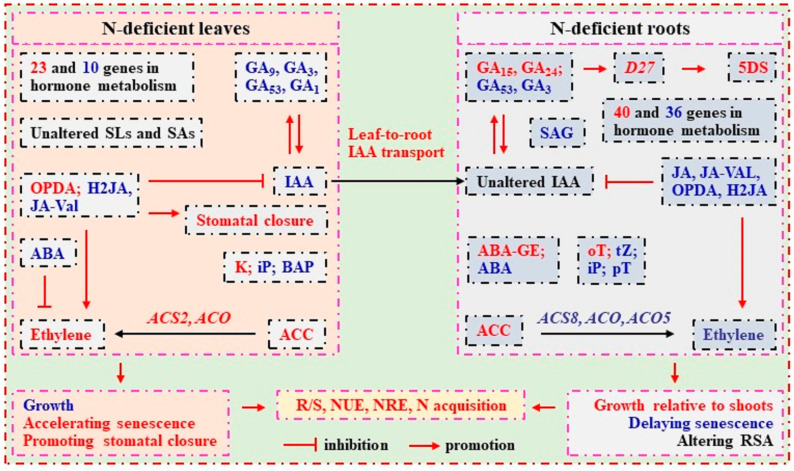
The schematic diagram of hormone responses to N deficiency in leaves and roots. Red, increase; Blue, decrease (refer to Lee and Yoon [116] and Tian et al. [101]).

Our results indicated that N deficiency reduced leaf growth and accelerated leaf senescence by lowering the concentrations of GAs (GA_9_, GA_3_, GA_53_, and GA_1_) and IAA, and increasing the concentrations of OPDA and the biosynthesis of ETH and SA. The increased formation of ETH in LN0 might be caused by decreased levels of ABA, and increased levels of ACC and OPDA. The increased level of OPDA in LN0 might lead to stomatal closure, thereby lowering transpiration water loss and conferring N deficiency tolerance. LN0 altered RSA, increased R/S, and delayed root senescence by lowering the levels of bioactive CKs, JA, OPDA, and ABA and the biosynthesis of ETH and SA, increasing the level of 5DS, and maintaining the homeostasis of IAA and GAs. The unchanged concentration of IAA in RN0 involved increased leaf-to-root IAA transport. Additionally, we obtained more DAHs and DTGs related to hormone metabolism in RN0 vs. RN15 (13 upregulated and 26 downregulated HRMs and 40 upregulated and 36 downregulated genes) than in LN0 vs. LN15 (16 upregulated and 16 downregulated HRMs and 23 upregulated and 10 downregulated genes). The different responses of leaf and root HRMs to N deficiency might be responsible for the increase in R/S and the modulations of RSA, thus improving NUE, NRE, and the ability to acquire N, and hence conferring citrus N deficiency tolerance (Figure 8). Therefore, our study provided the most comprehensive analysis of HRMs in response to N deficiency in citrus leaves and roots, and a foundation for the related study.

## Figures and Tables

**Table 1:** I Effects of N supply on mean (±SE, *n* = 3) concentrations (ng g^−1^ FW) of HRMs detected in *Citrus sinensis* leaves and roots.

HRMs	Leaves		Roots	
	LN0	LN15	RN0	RN15
Auxins (AUXs)				
Indole-3-acetic acid (IAA)	ND	6.67 ± 3.51	6.89 ± 0.88 a	9.05 ± 4.59 a
L-tryptophan (TRP)	5074 ± 212 a	2162 ± 651 b	5610 ± 1181 a	3710 ± 460 a
Tryptamine (TRA)	16.43 ± 7.91 a	0.32 ± 0.27 b	10.81 ± 0.43 a	4.03 ± 2.43 a
3-Indoleacetonitrile (IAN)	ND	0.32 ± 0.17	ND	0.39 ± 0.20
3-Indole acetamide (IAM)	ND	ND	ND	3.93 ± 0.37
2-oxindole-3-acetic acid (OxIAA)	ND	ND	13.80 ± 2.33 a	11.91 ± 0.44 a
Indole-3-butyric acid (IBA)	ND	ND	2.57 ± 1.29 a	4.03 ± 0.79 a
3-Indolepropionic acid (IPA)	ND	ND	0.41 ± 0.41 b	4.43 ± 0.18 a
Methyl indole-3-acetate (MEIAA)	0.51 ± 0.04 a	0.27 ± 0.09 a	1.70 ± 0.23 b	2.51 ± 0.05 a
N-(3-Indolylacetyl)-L-alanine (IAA-Ala)	ND	ND	ND	0.50 ± 0.03
N-(3-Indolylacetyl)-L-leucine (IAA-Leu)	ND	0.15 ± 0.15	ND	0.09 ± 0.09
Indole-3-acetyl-L-aspartic acid (IAA-Asp)	3.00 ± 3.00 a	16.81 ± 6.31 a	24.17 ± 3.59 a	23.76 ± 5.12 a
Indole-3-acetyl glycine (IAA-Gly)	18.73 ± 0.22 a	20.73 ± 3.31 a	4.61 ± 0.24 a	1.78 ± 0.35 b
Indole-3-acetyl glutamic acid (IAA-Glu)	1.86 ± 0.20 a	0.96 ± 0.18 b	2.56 ± 0.36 a	3.00 ± 1.30 a
N-(3-Indolylacetyl)-L-valine (IAA-Val)	2.59 ± 0.20 a	1.40 ± 0.17 b	1.11 ± 0.05 a	0.71 ± 0.15 a
Indole-3-acetyl-L-tryptophan (IAA-Trp)	0.66 ± 0.11 a	0.19 ± 0.19 a	ND	ND
Indole-3-acetyl-L-valine methyl ester (IAA-Val-Me)	ND	ND	0.11 ± 0.02	ND
Indole-3-acetyl-L-phenylalanne methyle ester (IAA-Phe-Me)	0.08 ± 0.01 a	0.09 ± 0.03 a	ND	ND
Indole-3-acetyl-L-leucine methyl ester (IAA-Leu-Me)	0.03 ± 0.03 a	0.09 ± 0.02 a	ND	ND
Indole-3-lactic acid (ILA)	102.12 ± 2.00 a	38.31 ± 2.55 b	6.87 ± 0.55 b	16.98 ± 2.65 a
Indole-3-carboxaldehyde (ICAld)	902.83 ± 31.46 a	1012.03 ± 48.26 a	21.10 ± 3.18 a	16.76 ± 1.97 a
Indole-3-carboxylic acid (ICA)	ND	0.67 ± 0.67	2.84 ± 0.68 a	1.43 ± 0.71 a
3-Indoleacrylic acid (IA)	7.34 ± 1.11	ND	0.42 ± 0.42 b	3.07 ± 0.21 a
Cytokinins (CKs)				
Isoprenoid CKs				
*trans*-Zeatin (tZ)	ND	ND	ND	0.13 ± 0.02
*trans*-Zeatin riboside (tZR)	0.13 ± 0.01 a	0.09 ± 0.01 a	0.26 ± 0.08 b	1.13 ± 0.11 a
*trans*-Zeatin-O-glucoside (tZOG)	32.00 ± 3.51 a	15.17 ± 2.08 b	5.43 ± 0.50 a	3.86 ± 0.15 b
*cis*-Zeatin (cZ)	ND	ND	0.01 ± 0.01 a	0.03 ± 0.01 a
*cis*-Zeatin riboside (cZR)	0.09 ± 0.01	ND	0.29 ± 0.03 b	0.40 ± 0.02 a
*cis*-Zeatin-O-glucoside riboside (cZROG)	0.84 ± 0.04 a	0.89 ± 0.06 a	0.32 ± 0.03 a	0.29 ± 0.01 a
*cis*-Zeatin-9-glucoside (cZ9G)	0.50 ± 0.09 a	0.66 ± 0.11 a	ND	ND
2-Methylthio-*cis*-zeatin (2MeScZ)	ND	ND	ND	0.005 ± 0.005
2-Methylthio-*cis*-zeatin riboside (2MeScZR)	0.14 ± 0.01 a	0.06 ± 0.00 b	0. 40 ± 0.00 a	0.39 ± 0.02 a
Dihydrozeatin ribonucleoside (DHZR)	0.54 ± 0.08	ND	ND	ND
Dihydrozeatin-7-glucoside (DHZ7G)	0.13 ± 0.01	ND	ND	ND
Dihydrozeatin-O-glucoside riboside (DHZROG)	0.03 ± 0.02 a	0.04 ± 0.00 a	ND	0.02 ± 0.01
*N*^6^-isopentenyladenine (iP)	ND	0.02 ± 0.01	0.10 ± 0.02 b	0.33 ± 0.05 a
*N*^6^-isopentenyladenosine (iPR)	ND	0.66 ± 0.14	0.60 ± 0.09 b	1.70 ± 0.33 a
*N*^6^-Isopentenyl-adenine-7-glucoside (iP7G)	1.46 ± 0.09 a	0.52 ± 0.03 b	0.14 ± 0.09 a	0.18 ± 0.09 a
*N*^6^-Isopentenyl-adenine-9-glucoside (iP9G)	0.68 ± 0.06 a	0.71 ± 0.02 a	ND	0.60 ± 0.36
2-Methylthio-*N*^6^-isopentenyladenine (2MeSiP)	ND	0.01 ± 0.01	ND	0.07 ± 0.03
2-Methylthio-*N*^6^-isopentenyladenosine (2MeSiPR)	ND	ND	11.42 ± 0.48 b	14.45 ± 0.20 a
Aromatic CKs				
Kinetin (K)	0.03 ± 0.03	ND	0.06 ± 0.01 a	0.07 ± 0.01 a
Kinetin riboside (KR)	ND	ND	0.02 ± 0.02	ND
Kinetin-9-glucoside (K9G)	ND	ND	0.53 ± 0.07 a	0.49 ± 0.25 a
6-Benzyladenine (BAP)	0.09 ± 0.09 b	0.50 ± 0.04 a	ND	ND
6-Benzyladenosine (BAPR)	ND	ND	0.07 ± 0.01 a	0.02 ± 0.02 b
*N*^6^-Benzyladenine-7-glucoside (BAP7G)	2.53 ± 0.17 b	3.87 ± 0.30 a	ND	ND
*N*^6^-Benzyladenine-9-glucoside (BAP9G)	ND	ND	ND	0.06 ± 0.06
meta-Topolin (mT)	0.20 ± 0.03 a	0.13 ± 0.07 a	0.14 ± 0.14 a	0.05 ± 0.03 a
meta-Topolin riboside (mTR)	0.04 ± 0.04	ND	ND	ND
meta-Topolin-9-glucoside (mT9G)	ND	ND	ND	0.34 ± 0.34
ortho-Topolin (oT)	ND	ND	0.06 ± 0.01	ND
ortho-Topolin riboside (oTR)	0.02 ± 0.02 a	0.11 ± 0.11 a	0.02 ± 0.02	ND
ortho-Topolin-9-glucoside (oT9G)	ND	ND	0.07 ± 0.04 a	0.22 ± 0.05 a
para-Topolin (pT)	0.16 ± 0.02 a	0.24 ± 0.06 a	ND	0.02 ± 0.02
4-[[(9-beta-D-Glucopyranosyl-9H-purin-6-yl)amino]methyl]phenol (pT9G)	2.03 ± 0.16 a	2.06 ± 0.10 a	1.41 ± 0.12 a	0.23 ± 0.23 b
Gibberellins (GAs)				
Gibberellin A_1_ (GA_1_)	ND	3.22 ± 3.22	124.03 ± 26.84 a	90.85 ± 9.87 a
Gibberellin A_3_ (GA_3_)	ND	0.34 ± 0.34	ND	0.43 ± 0.03
Gibberellin A_4_ (GA_4_)	ND	ND	0.57 ± 0.15 a	0.19 ± 0.19 a
Gibberellin A_9_ (GA_9_)	ND	0.10 ± 0.10	ND	ND
Gibberellin A_15_ (GA_15_)	ND	ND	0.82 ± 0.11 a	0.17 ± 0.06 b
Gibberellin A_19_ (GA_19_)	ND	ND	23.40 ± 0.52 a	19.6 ± 1.74 a
Gibberellin A_24_ (GA_24_)	ND	ND	9.50 ± 1.72 a	2.72 ± 0.16 b
Gibberellin A_53_ (GA_53_)	ND	0.19 ± 0.19	ND	0.68 ± 0.68
Jasmonates (JAs)				
Jasmonic acid (JA)	105.73 ± 7.29 a	106.53 ± 24.96 a	96.29 ± 5.85 b	324.89 ± 94.67 a
Methyl jasmonate (MEJA)	1.94 ± 0.24 a	3.60 ± 1.31 a	9.70 ± 0.48 a	9.99 ± 0.26 a
*cis*(+)-12-Oxophytodienoic acid (OPDA)	114.25 ± 22.46 a	46.74 ± 11.33 b	20.63 ± 1.86 b	71.30 ± 9.35 a
Dihydrojasmonic acid (H2JA)	1.12 ± 0.07 b	1.78 ± 0.06 a	0.66 ± 0.07 b	1.30 ± 0.17 a
Jasmonoyl-L-isoleucine (JA-ILE)	12.62 ± 2.57 a	20.86 ± 4.13 a	31.47 ± 2.02 a	53.04 ± 11.07 a
N-[(−)-Jasmonoyl]-(l)-phenalanine (JA-Phe)	0.53 ± 0.05 a	0.62 ± 0.10 a	0.25 ± 0.01 a	0.30 ± 0.06 a
N-[(−)-Jasmonoyl]-(L)-valine (JA-Val)	0.77 ± 0.03 b	1.56 ± 0.21 a	1.39 ± 0.00 b	5.39 ± 0.92 a
3-oxo-2-(2-(Z)-Pentenyl)cyclopentane-1-hexanoic acid (OPC-6)	27.68 ± 2.08 a	13.15 ± 7.13 a	ND	ND
3-oxo-2-(2-(Z)-Pentenyl) cyclopentane-1-butyric acid (OPC-4)	18.35 ± 1.78 a	21.04 ± 1.58 a	29.73 ± 2.58 a	28.06 ± 1.50 a
Abscisates (ABAs)				
Abscisic acid (ABA)	18.79 ± 3.09 b	48.52 ± 4.89 a	1.63 ± 0.24 b	5.99 ± 0.54 a
ABA-glucosyl ester (ABA-GE)	335.57 ± 42.09 a	232.35 ± 29.71 a	117.23 ± 6.16	ND
Salicylates (SAs)				
Salicylic acid (SA)	47.22 ± 1.29 a	45.16 ± 2.00 a	36.95 ± 3.76 a	30.94 ± 0.91 a
Salicylic acid 2-O-β-glucoside (SAG)	108.00 ± 27.26 a	37.28 ± 28.41 a	26.34 ± 2.09 b	47.42 ± 3.98 a
Strigolactones (SLs)				
5-Deoxystrigol (5DS)	36.07 ± 7.08 a	44.78 ± 6.38 a	4.95 ± 0.29 a	2.04 ± 0.22 b
(±)Strigol (ST)	95.33 ± 4.57 a	150.88 ± 28.61 a	208.35 ± 16.35 a	165.91 ± 8.36 a
Ethylene (ETH)				
1-Aminocyclopropanecarboxylic acid (ACC)	38.19 ± 0.63 a	24.23 ± 2.14 b	42.06 ± 4.09 a	13.59 ± 3.80 b

Different letters behind the values in the same row for the same tissue (leaves or tissue) represent significant differences at *p* < 0.05. ND, not detected. An HRM was considered downregulated or upregulated when it had both a relative change of less or more than 1 and a *p* < 0.05. Also, an HRM was considered upregulated or downregulated when it was detected only in RN0 (LN0) or RN15 (LN15), respectively, in RN0 vs. RN15 (LN0 vs. LN15).

**Table 2:** Formula of macronutrients.

N Levels (mM)	Macronutrients (mM)							
	K_2_SO_4_	KH_2_PO_4_	KNO_3_	MgSO_4_	Ca(NO_3_)_2_	(NH_4_)_2_SO_4_	NH_4_Cl	CaCl_2_
0	2.5	1	0	2	0	0	0	5
15	1.25	1	2.5	2	2.5	1.25	5	2.5

## Data Availability

RNA-Seq data for leaves and roots were deposited in an NCBI database with the SRA accession numbers PRJNA878600 (https://www.ncbi.nlm.nih.gov/bioproject/?term=PRJNA878600, accessed on 28 April 2024) and PRJNA890033 (https://www.ncbi.nlm.nih.gov/bioproject/?term=PRJNA890033, accessed on 28 April 2024), respectively. Data are archived in L.-S. Chen’s lab and available upon request.

## Supplementary Materials

The following supporting information can be downloaded at: https://www.mdpi.com/article/10.3390/plants13141925/s1, Table S1: List of 88 hormones tested in *Citrus sinensis* roots and leaves; Table S2: Differentially transcribed genes (DTGs) related to hormone metabolism identified in leaves of 0 mM N-treated seedlings (LN0) vs. leaves of 15 mM N-treated seedlings (LN15) and roots of 0 mM N-treated seedlings (RN0) vs. roots of 15 mM N-treated seedlings (RN15).

## References

[1] Camut L., Gallova B., Jilli L., Sirlin-Josserand M., Carrera E., Sakvarelidze-Achard L., Ruffel S., Krouk G., Thomas S.G., Hedden P. (2021). Nitrate signaling promotes plant growth by upregulating gibberellin biosynthesis and destabilization of DELLA proteins. Curr. Biol..

[2] Huang W.-T., Zheng Z.-C., Hua D., Chen X.-F., Zhang J., Chen H.-H., Ye X., Guo J.-X., Yang L.-T., Chen L.-S. (2022). Adaptive responses of carbon and nitrogen metabolisms to nitrogen-deficiency in *Citrus sinensis* seedlings. BMC Plant Biol..

[3] Wu S., Liang S., Hu C., Tan Q., Zhang J., Dong Z. (2022). Ecological region division of soil based supplementary fertilization and decrement fertilization in China citrus orchards. J. Huazhong Agri. Univ..

[4] Hua D., Chen W.-S., Rao R.-Y., Chen X.-F., Chen H.-H., Lai N.-W., Yang L.-T., Ye X., Chen L.-S. (2024). Nitrogen-deficient *Citrus sinensis* leaves and roots can keep high abilities to scavenge reactive oxygen species and methylglyoxal, and protect them against oxidative damage. Sci. Horti..

[5] Conesa C.M., Saez A., Navarro-Neila S., de Lorenzo L., Hunt A.G., Sepúlveda E.B., Baigorri R., Garcia-Mina J.M., Zamarreño A.M., Sacristán S. (2020). Alternative polyadenylation and salicylic acid modulate root responses to low nitrogen availability. Plants.

[6] Lai Y.-H., Peng M.-Y., Rao R.-Y., Chen W.-S., Huang W.-T., Ye X., Yang L.-T., Chen L.-S. (2023). An integrated analysis of metabolome, transcriptome, and physiology revealed the molecular and physiological response of *Citrus sinensis* roots to prolonged nitrogen deficiency. Plants.

[7] Santner A., Calderon-Villalobos L.I., Estelle M. (2009). Plant hormones are versatile chemical regulators of plant growth. Nat. Chem. Biol..

[8] Drew M.C., He C.J., Morgan P.W. (1989). Decreased ethylene biosynthesis, and induction of aerenchyma, by nitrogen-starvation or phosphate-starvation in adventitious roots of *Zea mays*. Plant Physiol..

[9] Sun X., Chen F., Yuan L., Mi G. (2020). The physiological mechanism underlying root elongation in response to nitrogen deficiency in crop plants. Planta.

[10] Sun H., Guo X., Zhu X., Gu P., Zhang W., Tao W., Wang D., Wu Y., Zhao Q., Xu G. (2023). Strigolactone and gibberellin signaling coordinately regulate metabolic adaptations to changes in nitrogen availability in rice. Mol. Plant.

[11] Tian Q., Chen F., Liu J., Zhang F., Mi G. (2008). Inhibition of maize root growth by high nitrate supply is correlated with reduced IAA levels in roots. J. Plant Physiol..

[12] Sun X., Chen H., Wang P., Chen F., Yuan L., Mi G. (2020). Low nitrogen induces root elongation *via* auxin-induced acid growth and auxin-regulated target of rapamycin (TOR) pathway in maize. J. Plant Physiol..

[13] Werner T., Motyka V., Laucou V., Smets R., Van Onckelen H., Schmülling T. (2003). Cytokinin-deficient transgenic *Arabidopsis* plants show multiple developmental alterations indicating opposite functions of cytokinins in the regulation of shoot and root meristem activity. Plant Cell.

[14] Mardanov A., Samedovam A., Shirvany T., Box J.E. (1998). Root-shoot relationships in plant adaptation to nitrogen deficiency. Root Demographics and Their Efficiencies in Sustainable Agriculture, Grasslands and Forest Ecosystems.

[15] Chen Q., Sun J., Zhai Q., Zhou W., Qi L., Xu L., Wang B., Chen R., Jiang H., Qi J. (2011). The basic helix-loop-helix transcription factor MYC2 directly represses PLETHORA expression during jasmonate-mediated modulation of the root stem cell niche in *Arabidopsis*. Plant Cell.

[16] Brookbank B.P., Patel J., Gazzarrini S., Nambara E. (2021). Role of basal ABA in plant growth and development. Genes.

[17] Rivas-San Vicente M., Plasencia J. (2011). Salicylic acid beyond defence: Its role in plant growth and development. J. Exp. Bot..

[18] Paponov M., Arakelyan A., Dobrev P.I., Verheul M.J., Paponov I.A. (2021). Nitrogen deficiency and synergism between continuous light and root ammonium supply modulate distinct but overlapping patterns of phytohormone composition in xylem sap of tomato plants. Plants.

[19] Wang Y., Yao Q., Zhang Y., Zhang Y., Xing J., Yang B., Mi G., Li Z., Zhang M. (2020). The Role of gibberellins in regulation of nitrogen uptake and physiological traits in maize responding to nitrogen availability. Int. J. Mol. Sci..

[20] Lv X., Zhang Y., Hu L., Zhang Y., Zhang B., Xia H., Du W., Kong L. (2021). Low-nitrogen stress stimulates lateral root initiation and nitrogen assimilation in wheat: Roles of phytohormone signaling. J. Plant Growth Regul..

[21] Sun H., Tao J., Gu P., Xu G., Zhang Y. (2016). The role of strigolactones in root development. Plant Signal. Behav..

[22] Soliman S., Wang Y., Han Z., Pervaiz T., El-Kereamy A. (2022). Strigolactones in plants and their interaction with the ecological microbiome in response to abiotic stress. Plants.

[23] Oka M., Shimoda Y., Sato N., Inoue J., Yamazaki T., Shimomura N., Fujiyama H. (2012). Abscisic acid substantially inhibits senescence of cucumber plants (*Cucumis sativus*) grown under low nitrogen conditions. J. Plant Physiol..

[24] Jia X., Wang Q., Ye Y., Li T., Sun X., Huo L., Gong X., Ma F. (2022). *MdATG5a* positively regulates nitrogen uptake under low nitrogen conditions by enhancing the accumulation of flavonoids and auxin in apple roots. Environ. Exp. Bot..

[25] Peng M.-Y., Ren Q.-Q., Lai Y.-H., Zhang J., Chen H.-H., Guo J., Yang L.-T., Chen L.-S. (2023). Integration of physiology, metabolome and transcriptome for understanding of the adaptive strategies to long-term nitrogen deficiency in *Citrus sinensis* leaves. Sci. Horti..

[26] Lim P.O., Kim H.J., Nam H.G. (2007). Leaf senescence. Annu. Rev. Plant Biol..

[27] Gören-Sağlam N., Harrison E., Breeze E., Öz G., Buchanan-Wollaston V. (2020). Analysis of the impact of indole-3-acetic acid (IAA) on gene expression during leaf senescence in *Arabidopsis thaliana*. Physiol. Mol. Biol. Plants.

[28] Jibran R., Hunter D.A., Dijkwel P.P. (2013). Hormonal regulation of leaf senescence through integration of developmental and stress signals. Plant Mol. Biol..

[29] Zakari S.A., Asad M.A.U., Han Z., Zhao Q., Cheng F. (2020). Relationship of nitrogen deficiency-induced leaf senescence with ROS generation and ABA concentration in rice flag leaves. J. Plant Growth Regul..

[30] Pascual L.S., Mittler R., Sinha R., Peláez-Vico M.Á., María F., López-Climent M.F., Vives-Peris V., Gómez-Cadenas A., Zandalinas A.I. (2023). Jasmonic acid is required for tomato acclimation to multifactorial stress combination. Environ. Exp. Bot..

[31] Stoparić G., Maksimović I. (2008). The effect of cytokinins on the concentration of hydroxyl radicals and the intensity of lipid peroxidation in nitrogen deficient wheat. Cereal Res. Commun..

[32] Iqbal N., Umar S., Khan N.A. (2015). Nitrogen availability regulates proline and ethylene production and alleviates salinity stress in mustard (*Brassica juncea*). J. Plant Physiol..

[33] Squeri C., Miras-Moreno B., Gatti M., Garavani A., Poni S., Lucini L., Trevisan M. (2021). Gas exchange, vine performance and modulation of secondary metabolism in *Vitis vinifera* L. cv Barbera following long-term nitrogen deficit. Planta.

[34] Schmelz E.A., Alborn H.T., Engelberth J., Tumlinson J.H. (2003). Nitrogen deficiency increases volicitin-induced volatile emission, jasmonic acid accumulation, and ethylene sensitivity in maize. Plant Physiol..

[35] Chen J., Liu S., Zhang S., Ge C., Shen Q., Ma H., Zhang X., Dong H., Zhao X., Pang C. (2021). Nitrogen modulates cotton root morphology by affecting abscisic acid (ABA) and salicylic acid (SA) content. Arch. Agron. Soil Sci..

[36] Tian Q.Y., Sun P., Zhang W.H. (2009). Ethylene is involved in nitrate-dependent root growth and branching in *Arabidopsis thaliana*. New Phytol..

[37] Huang W.-T., Xie Y.-Z., Chen X.-F., Zhang J., Chen H.-H., Ye X., Guo J., Yang L.-T., Chen L.-S. (2021). Growth, mineral nutrients, photosynthesis and related physiological parameters of citrus in response to nitrogen-deficiency. Agronomy.

[38] Sakakibara H. (2006). Cytokinins: Activity, biosynthesis, and translocation. Annu. Rev. Plant Biol..

[39] Mano Y., Nemoto K. (2012). The pathway of auxin biosynthesis in plants. J. Exp. Bot..

[40] Woodward A.W., Bartel B. (2005). Auxin: Regulation, action, and interaction. Ann. Bot..

[41] Li L., He Y., Wang Y., Zhao S., Chen X., Ye T., Wu Y., Wu Y. (2015). *Arabidopsis* PLC2 is involved in auxin-modulated reproductive development. Plant J..

[42] Zhao Y. (2012). Auxin biosynthesis: A simple two-step pathway converts tryptophan to indole-3-acetic acid in plants. Mol. Plant.

[43] Seo M., Akaba S., Oritani T., Delarue M., Bellini C., Caboche M., Koshiba T. (1998). Higher activity of an aldehyde oxidase in the auxin-overproducing superroot1 mutant of *Arabidopsis thaliana*. Plant Physiol..

[44] Ding C., Lin X., Zuo Y., Yu Z., Baerson S.R., Pan Z., Zeng R., Song Y. (2021). Transcription factor OsbZIP49 controls tiller angle and plant architecture through the induction of indole-3-acetic acid-amido synthetases in rice. Plant J..

[45] Tognetti V.B., Mühlenbock P., Van Breusegem F. (2012). Stress homeostasis—The redox and auxin perspective. Plant Cell Environ..

[46] Barlier I., Kowalczyk M., Marchant A., Ljung K., Bhalerao R., Bennett M., Sandberg G., Bellini C. (2000). The SUR2 gene of *Arabidopsis thaliana* encodes the cytochrome P450 CYP83B1, a modulator of auxin homeostasis. Proc. Natl. Acad. Sci. USA.

[47] Bartel B., Fink G.R. (1995). ILR1, an amidohydrolase that releases active indole-3-acetic acid from conjugates. Science.

[48] Korasick D.A., Enders T.A., Strader L.C. (2013). Auxin biosynthesis and storage forms. J. Exp. Bot..

[49] Rampey R.A., LeClere S., Kowalczyk M., Ljung K., Sandberg G., Bartel B. (2004). A family of auxin-conjugate hydrolases that contributes to free indole-3-acetic acid levels during *Arabidopsis* germination. Plant Physiol..

[50] Yang Y., Xu R., Ma C.J., Vlot A.C., Klessig D.F., Pichersky E. (2008). Inactive methyl indole-3-acetic acid ester can be hydrolyzed and activated by several esterases belonging to the AtMES esterase family of *Arabidopsis*. Plant Physiol..

[51] Strader L.C., Culler A.H., Cohen J.D., Bartel B. (2010). Conversion of endogenous indole-3-butyric acid to indole-3-acetic acid drives cell expansion in *Arabidopsis* seedlings. Plant Physiol..

[52] Grira M., Prinsen E., Werbrouck S.P.O. (2023). The effect of topophysis on the *in vitro* development of *Handroanthus guayacan* and on its metabolism of meta-topolin riboside. Plants.

[53] Hirose N., Takei K., Kuroha T., Kamada-Nobusada T., Hayashi H., Sakakibara H. (2008). Regulation of cytokinin biosynthesis, compartmentalization and translocation. J. Exp. Bot..

[54] Rodo A.P., Brugière N., Vankova R., Malbeck J., Olson J.M., Haines S.C., Martin R.C., Habben J.E., Mok D.W., Mok M.C. (2008). Over-expression of a zeatin O-glucosylation gene in maize leads to growth retardation and tasselseed formation. J. Exp. Bot..

[55] Wang Q., Zhu Y., Zou X., Li F., Zhang J., Kang Z., Li X., Yin C., Lin Y. (2020). Nitrogen deficiency-induced decrease in cytokinins content promotes rice seminal root growth by promoting root meristem cell proliferation and cell elongation. Cells.

[56] Tian Q., Chen F., Zhang F., Mi G. (2005). Possible involvement of cytokinin in nitrate-mediated root growth in maize. Plant Soil.

[57] Gan S.S., Amasino R.M. (1995). Inhibition of leaf senescence by autoregulated production of cytokinin. Science.

[58] Binenbaum J., Weinstain R., Shani E. (2018). Gibberellin localization and transport in plants. Trends Plant Sci..

[59] Yamaguchi S. (2008). Gibberellin metabolism and its regulation. Annu. Rev. Plant Biol..

[60] Qi W., Sun F., Wang Q., Chen M., Huang Y., Feng Y.Q., Luo X., Yang J. (2011). Rice ethylene-response AP2/ERF factor OsEATB restricts internode elongation by down-regulating a gibberellin biosynthetic gene. Plant Physiol..

[61] Swain S.M., Singh D.P., Helliwell C.A., Poole A.T. (2005). Plants with increased expression of *ent*-kaurene oxidase are resistant to chemical inhibitors of this gibberellin biosynthesis enzyme. Plant Cell Physiol..

[62] Achard P., Baghour M., Chapple A., Hedden P., Van Der Straeten D., Genschik P., Moritz T., Harberd N.P. (2007). The plant stress hormone ethylene controls floral transition *via* DELLA-dependent regulation of floral meristem-identity genes. Proc. Natl. Acad. Sci. USA.

[63] Fleet C.M., Yamaguchi S., Hanada A., Kawaide H., David C.J., Kamiya Y., Sun T.P. (2003). Overexpression of *AtCPS* and *AtKS* in *Arabidopsis* confers increased *ent*-kaurene production but no increase in bioactive gibberellins. Plant Physiol..

[64] Huang S., Raman A.S., Ream J.E., Fujiwara H., Cerny R.E., Brown S.M. (1998). Overexpression of 20-oxidase confers a gibberellin-overproduction phenotype in *Arabidopsis*. Plant Physiol..

[65] Radi A., Lange T., Niki T., Koshioka M., Lange M.J.P. (2006). Ectopic expression of pumpkin gibberellin oxidases alters gibberellin biosynthesis and development of transgenic *Arabidopsis* plants. Plant Physiol..

[66] Gallego-Giraldo L., Ubeda-Tomás S., Gisbert C., García-Martínez J.L., Moritz T., López-Díaz I. (2008). Gibberellin homeostasis in tobacco is regulated by gibberellin metabolism genes with different gibberellin sensitivity. Plant Cell Physiol..

[67] Vidal A.M., Gisbert C., Talón M., Primo-Millo E., López-Díaz I., García-Martínez J.L. (2001). The ectopic overexpression of a citrus gibberellin 20-oxidase enhances the non-13-hydroxylation pathway of gibberellin biosynthesis and induces an extremely elongated phenotype in tobacco. Physiol. Plant..

[68] Wang F., Yoshida H., Matsuoka M. (2021). Making the ‘Green Revolution’ truly green: Improving crop nitrogen use efficiency. Plant Cell Physiol..

[69] Kou E., Huang X., Zhu Y., Su W., Liu H., Sun G., Chen R., Hao Y., Song S. (2021). Crosstalk between auxin and gibberellin during stalk elongation in flowering Chinese cabbage. Sci. Rep..

[70] Hu T., Hu Z., Zeng H., Qv X., Chen G. (2015). Tomato lipoxygenase D involved in the biosynthesis of jasmonic acid and tolerance to abiotic and biotic stress in tomato. Plant Biotechnol. Rep..

[71] Hazman M., Hause B., Eiche E., Nick P., Riemann M. (2015). Increased tolerance to salt stress in OPDA-deficient rice ALLENE OXIDE CYCLASE mutants is linked to an increased ROS-scavenging activity. J. Exp. Bot..

[72] Xiong X.P., Sun S.C., Zhu Q.H., Zhang X.Y., Li Y.J., Liu F., Xue F., Sun J. (2021). The cotton lignin biosynthetic gene Gh4CL30 regulates lignification and phenolic content and contributes to *Verticillium* wilt resistance. Mol. Plant Microbe Interact..

[73] Kienow L., Schneider K., Bartsch M., Stuible H.P., Weng H., Miersch O., Wasternack C., Kombrink E. (2008). Jasmonates meet fatty acids: Functional analysis of a new acyl-coenzyme A synthetase family from *Arabidopsis thaliana*. J. Exp. Bot..

[74] Savchenko T., Kolla V.A., Wang C.Q., Nasafi Z., Hicks D.R., Phadungchob B., Chehab W.E., Brandizzi F., Froehlich J., Dehesh K. (2014). Functional convergence of oxylipin and abscisic acid pathways controls stomatal closure in response to drought. Plant Physiol..

[75] Zhang Y., Turner J.G. (2008). Wound-induced endogenous jasmonates stunt plant growth by inhibiting mitosis. PLoS ONE.

[76] Mueller S., Hilbert B., Dueckershoff K., Roitsch T., Krischke M., Mueller M.J., Berger S. (2008). General detoxification and stress responses are mediated by oxidized lipids through TGA transcription factors in *Arabidopsis*. Plant Cell.

[77] Wu X., Ding C., Baerson S.R., Lian F., Lin X., Zhang L., Wu C., Hwang S.Y., Zeng R., Song Y. (2019). The roles of jasmonate signalling in nitrogen uptake and allocation in rice (*Oryza sativa* L.). Plant Cell Environ..

[78] Chu L.L., Yan Z., Sheng X.X., Liu H.Q., Wang Q.Y., Zeng R.F., Hu C.G., Zhang J.Z. (2023). Citrus ACC synthase *CiACS4* regulates plant height by inhibiting gibberellin biosynthesis. Plant Physiol..

[79] Wi S.J., Park K.Y. (2002). Antisense expression of carnation cDNA encoding ACC synthase or ACC oxidase enhances polyamine content and abiotic stress tolerance in transgenic tobacco plants. Mol. Cells.

[80] Zheng D., Han X., An Y.I., Guo H., Xia X., Yin W. (2013). The nitrate transporter NRT2.1 functions in the ethylene response to nitrate deficiency in *Arabidopsis*. Plant Cell Environ..

[81] Zhang Y., Wang Y., Liu C., Ye D., Ren D., Li Z., Zhang M. (2022). Ethephon reduces maize nitrogen uptake but improves nitrogen utilization in *Zea mays* L.. Front. Plant Sci..

[82] Léran S., Muños S., Brachet C., Tillard P., Gojon A., Lacombe B. (2013). *Arabidopsis* NRT1.1 is a bidirectional transporter involved in root-to-shoot nitrate translocation. Mol. Plant.

[83] Rauf M., Arif M., Fisahn J., Xue G.P., Balazadeh S., Mueller-Roeber B. (2013). NAC transcription factor speedy hyponastic growth regulates flooding-induced leaf movement in *Arabidopsis*. Plant Cell.

[84] Varsani S., Grover S., Zhou S., Koch K.G., Huang P.C., Kolomiets M.V., Williams W.P., Heng-Moss T., Sarath G., Luthe D.S. (2019). 12-oxo-phytodienoic acid acts as a regulator of maize defense against corn leaf aphid. Plant Physiol..

[85] Frey A., Audran C., Marin E., Sotta B., Marion-Poll A. (1999). Engineering seed dormancy by the modification of zeaxanthin epoxidase gene expression. Plant Mol. Biol..

[86] González-Guzmán M., Abia D., Salinas J., Serrano R., Rodríguez P.L. (2004). Two new alleles of the abscisic aldehyde oxidase 3 gene reveal its role in abscisic acid biosynthesis in seeds. Plant Physiol..

[87] Seo M., Koiwai H., Akaba S., Komano T., Oritani T., Kamiya Y., Koshiba T. (2000). Abscisic aldehyde oxidase in leaves of *Arabidopsis thaliana*. Plant J..

[88] Bao Y., Song W.M., Pan J., Jiang C.M., Srivastava R., Li B., Zhu L.Y., Su H.Y., Gao X.S., Liu H. (2016). Overexpression of the NDR1/HIN1-Like gene *NHL6* modifies seed germination in response to abscisic acid and abiotic stresses in Arabidopsis. PLoS ONE.

[89] Yang S.H., Zeevaart J.A. (2006). Expression of ABA 8′-hydroxylases in relation to leaf water relations and seed development in bean. Plant J..

[90] Lee W.J., Truong H.A., Trịnh C.S., Kim J.H., Lee S., Hong S.W., Lee H. (2020). NITROGEN RESPONSE DEFICIENCY 1-mediated CHL1 induction contributes to optimized growth performance during altered nitrate availability in *Arabidopsis*. Plant J..

[91] Chen K., Li G.J., Bressan R.A., Song C.P., Zhu J.K., Zhao Y. (2020). Abscisic acid dynamics, signaling, and functions in plants. J. Integr. Plant Biol..

[92] Watanabe S., Sato M., Sawada Y., Tanaka M., Matsui A., Kanno Y., Hirai M.Y., Seki M., Sakamoto A., Seo M. (2018). *Arabidopsis* molybdenum cofactor sulfurase ABA3 contributes to anthocyanin accumulation and oxidative stress tolerance in ABA-dependent and independent ways. Sci. Rep..

[93] Tan B.C., Schwartz S.H., Zeevaart J.A.D., McCarty D.R. (1997). Genetic control of abscisic acid biosynthesis in maize. Proc. Natl. Acad. Sci. USA.

[94] Dodd I.C. (2003). Leaf area development of ABA-deficient and wild-type peas at two levels of nitrogen supply. Funct. Plant Biol..

[95] Shi H., Ma W., Song J., Lu M., Rahman S.U., Bui T.T.X., Vu D.D., Zheng H., Wang J., Zhang Y. (2017). Physiological and transcriptional responses of *Catalpa bungei* to drought stress under sufficient- and deficient-nitrogen conditions. Tree Physiol..

[96] Xiong Q., Tang G., Zhong L., He H., Chen X. (2018). Response to nitrogen deficiency and compensation on physiological characteristics, yield formation, and nitrogen utilization of rice. Front. Plant Sci..

[97] Hussain A., Black C.R., Taylor I.B., Roberts J.A. (2000). Does an antagonistic relationship between ABA and ethylene mediate shoot growth when tomato (*Lycopersicon esculentum* Mill.) plants encounter compacted soil?. Plant Cell Environ..

[98] Van Volkenburgh E., Davies W.J. (1983). Inhibition of light-stimulated leaf expansion by abscisic acid. J. Exp. Bot..

[99] Arite T., Umehara M., Ishikawa S., Hanada A., Maekawa M., Yamaguchi S., Kyozuka J. (2009). *d14*, a strigolactone-insensitive mutant of rice, shows an accelerated outgrowth of tillers. Plant Cell Physiol..

[100] Yoneyama K., Xie X., Kusumoto D., Sekimoto H., Sugimoto Y., Takeuchi Y., Yoneyama K. (2007). Nitrogen deficiency as well as phosphorus deficiency in sorghum promotes the production and exudation of 5-deoxystrigol, the host recognition signal for arbuscular mycorrhizal fungi and root parasites. Planta.

[101] Tian Z., Zhang Y., Zhu L., Jiang B., Wang H., Gao R., Friml J., Xiao G. (2022). Strigolactones act downstream of gibberellins to regulate fiber cell elongation and cell wall thickness in cotton (*Gossypium hirsutum*). Plant Cell.

[102] Xia Y., Suzuki H., Borevitz J., Blount J., Guo Z., Patel K., Dixon R.A., Lamb C. (2004). An extracellular aspartic protease functions in *Arabidopsis* disease resistance signaling. EMBO J..

[103] Zhang Y., Li X. (2019). Salicylic acid: Biosynthesis, perception, and contributions to plant immunity. Curr. Opin. Plant Biol..

[104] Cui H., Gobbato E., Kracher B., Qiu J., Bautor J., Parker J.E. (2017). A core function of EDS1 with PAD4 is to protect the salicylic acid defense sector in *Arabidopsis* immunity. New Phytol..

[105] Vogelmann K., Drechsel G., Bergler J., Subert C., Philippar K., Soll J., Engelmann J.C., Engelsdorf T., Voll L.M., Hoth S. (2012). Early senescence and cell death in *Arabidopsis saul1* mutants involves the *PAD4*-dependent salicylic acid pathway. Plant Physiol..

[106] Ding P., Ding Y. (2020). Stories of salicylic acid: A plant defense hormone. Trends Plant Sci..

[107] Zhang K., Halitschke R., Yin C., Liu C.J., Gan S.S. (2013). Salicylic acid 3-hydroxylase regulates *Arabidopsis* leaf longevity by mediating salicylic acid catabolism. Proc. Natl. Acad. Sci. USA.

[108] Song J.T., Koo Y.J., Seo H.S., Kim M.C., Choi Y.D., Kim J.H. (2008). Overexpression of *AtSGT1*, an *Arabidopsis* salicylic acid glucosyltransferase, leads to increased susceptibility to *Pseudomonas syringae*. Phytochemistry.

[109] Yang T.-Y., Cai L.-Y., Qi Y.-P., Yang L.-T., Lai N.-W., Chen L.-S. (2019). Increasing nutrient solution pH alleviated aluminum-induced inhibition of growth and impairment of photosynthetic electron transport chain in *Citrus sinensis* seedlings. BioMed Res. Int..

[110] Wu B.-S., Lai Y.-H., Peng M.-Y., Ren Q.-Q., Lai N.-W., Wu J., Huang Z.-R., Yang L.-T., Chen L.-S. (2022). Elevated pH-mediated mitigation of aluminum-toxicity in sweet orange (*Citrus sinensis*) roots involved the regulation of energy-rich compounds and phytohormones. Environ. Pollut..

[111] Wang X., Wei X., Zhao W., Li X., Dong S. (2024). Elucidating hormone transduction and the protein response of soybean roots to drought stress based on ultra performance liquidchromatography–tandem mass spectrometry and four-dimensional data-independent acquisition. Environ. Exp. Bot..

[112] Pan X., Welti R., Wang X. (2010). Quantitative analysis of major plant hormones in crude plant extracts by high-performance liquid chromatography-mass spectrometry. Nat. Protoc..

[113] Šimura J., Antoniadi I., Široká J., Tarkowská D., Strnad M., Ljung K., Novák O. (2018). Plant Hormonomics: Multiple phytohormone profiling by targeted metabolomics. Plant Physiol..

[114] Floková K., Tarkowská D., Miersch O., Strnad M., Wasternack C., Novák O. (2014). UHPLC-MS/MS based target profiling of stress-induced phytohormones. Phytochemistry.

[115] Guo P., Qi Y.-P., Huang W.-L., Yang L.-T., Huang Z.-R., Lai N.-W., Chen L.-S. (2018). Aluminum-responsive genes revealed by RNA-Seq and related physiological responses in leaves of two citrus species with contrasting aluminum-tolerance. Ecotoxicol. Environ. Saf..

[116] Lee H.Y., Yoon G.M. (2018). Regulation of ethylene biosynthesis by phytohormones in etiolated rice (*Oryza sativa* L.) seedlings. Mol. Cells.

