# Supplementary material for: Adaptive Responses of Hormones to Nitrogen Deficiency in Citrus sinensis Leaves and Roots
Source: Plants (Basel). 2024 Jul 12;13(14):1925. doi: 10.3390/plants13141925 (PMC11280038; doi:10.3390/plants13141925)
Supplement: Supplementary file 1 [file plants-13-01925-s001.zip › plants-3084648-Table S2.pdf]

Table S2. Differentially transcribed genes (DTGs) related to hormone metabolism identified in leaves of 0 mM N-treated seedlings (LN0) vs. leaves of 15 mM N-treated seedlings (LN15) and roots of 0 mM N-treated seedlings (RN0) vs. roots of 15 mM N-treated seedlings (RN15).

Accession No.	KEGG	Swissprot (KOG)	Log_2(fold change)	
			LN0 vs. LN15	RN0 vs. RN15
Auxin biosynthetic process [GO:0009851; P = 0.1348 (LN0 vs. LN15) or 0.1197 (RN0 vs. RN15)]				
Cs1g23870	K11816 indole-3-pyruvate monooxygenase [EC:1.14.13.168] | (RefSeq) indole-3-pyruvate monooxygenase YUCCA2-like (A)	Indole-3-pyruvate monooxygenase YUCCA2; EC=1.14.13.168; Flavin-containing monooxygenase YUCCA2 (At4g13260)	1.995	
Cs2g17930	K01658 anthranilate synthase component II [EC:4.1.3.27] | (RefSeq) anthranilate synthase beta subunit 2, chloroplastic-like (A)	Anthranilate synthase beta subunit 1, chloroplastic; EC=4.1.3.27; Anthranilate synthase component 2-1; Anthranilate synthase, glutamine amidotransferase component 2-1; Protein TRYPTOPHAN BIOSYNTHESIS 4; Protein WEAK ETHYLENE INSENSITIVE 7 (At1g25220)		-1.192
Cs3g07330	K20617 cytochrome P450 family 71 subfamily A | (RefSeq) cytochrome P450 71A1-like (A)	Cytochrome P450 83B1; EC=1.14.14.45 {ECO:0000269|PubMed:12970475}; Protein ALTERED TRYPTOPHAN REGULATION 4; Protein RED ELONGATED 1; Protein SUPERROOT 2 (At4g31500)	-1.986	
Cs3g25510	--	Protein WALLS ARE THIN 1 (At1g75500)		-1.694
Cs3g25780	K20617 cytochrome P450 family 71 subfamily A | (RefSeq) cytochrome P450 71A9-like (A)	Cytochrome P450 83B1; EC=1.14.14.45 {ECO:0000269|PubMed:12970475}; Protein ALTERED TRYPTOPHAN REGULATION 4; Protein RED ELONGATED 1; Protein SUPERROOT 2 (At4g31500)	2.075	
Cs5g25880	K20617 cytochrome P450 family 71 subfamily A | (RefSeq) cytochrome P450 71A9-like (A)	Cytochrome P450 83B1; EC=1.14.14.45 {ECO:0000269|PubMed:12970475}; Protein ALTERED TRYPTOPHAN REGULATION 4; Protein RED ELONGATED 1; Protein SUPERROOT 2 (At4g31500)		3.612
Cs5g25920	K20617 cytochrome P450 family 71 subfamily A | (RefSeq) cytochrome P450 71A9-like (A)	Cytochrome P450 83B1; EC=1.14.14.45 {ECO:0000269|PubMed:12970475}; Protein ALTERED TRYPTOPHAN REGULATION 4; Protein RED ELONGATED 1; Protein SUPERROOT 2 (At4g31500)		3.960
Cs5g32440	K11816 indole-3-pyruvate monooxygenase [EC:1.14.13.168] | (RefSeq) probable indole-3-pyruvate monooxygenase YUCCA10 (A)	Probable indole-3-pyruvate monooxygenase YUCCA10; EC=1.14.13.168; Flavin-containing monooxygenase YUCCA10 (At1g48910)		-1.757
Cs5g34410	K11816 indole-3-pyruvate monooxygenase [EC:1.14.13.168] | (RefSeq) probable indole-3-pyruvate monooxygenase YUCCA5 (A)	Probable indole-3-pyruvate monooxygenase YUCCA8; EC=1.14.13.168; Flavin-containing monooxygenase YUCCA8 (At4g28720)		-1.767
Cs7g14760	K05857 phosphatidylinositol phospholipase C, delta [EC:3.1.4.11] | (RefSeq) phosphoinositide phospholipase C 2-like (A)	Phosphoinositide phospholipase C 2; EC=3.1.4.11; Phosphoinositide phospholipase PLC2; AtPLC2; PI-PLC2 (At3g08510)	2.283	
Cs7g22090	--	Protein SHOOT GRAVITROPISM 5; Protein indeterminate-domain 15 {ECO:0000303|PubMed:16784536}; AtIDD15 (At3g48550)		-2.999

Cs8g13760	K09842 abscisic-aldehyde oxidase [EC:1.2.3.14] | (RefSeq) AAO3; abscisic-aldehyde oxidase (A)	Indole-3-acetaldehyde oxidase; IAA oxidase; EC=1.2.3.7; Aldehyde oxidase 1; AO-1; AtAO-1; AtAO1 (At5g20960)		2.775
Cs8g13770	K09842 abscisic-aldehyde oxidase [EC:1.2.3.14] | (RefSeq) indole-3-acetaldehyde oxidase-like (A)	Indole-3-acetaldehyde oxidase; IAA oxidase; EC=1.2.3.7; Aldehyde oxidase 1; AO-1; AtAO-1; AtAO1 (At5g20960)		1.065
Cs8g20260	K05857 phosphatidylinositol phospholipase C, delta [EC:3.1.4.11] | (RefSeq) phosphoinositide phospholipase C 2-like (A)	Phosphoinositide phospholipase C 2; EC=3.1.4.11; Phosphoinositide phospholipase PLC2; AtPLC2; PI-PLC2 (At3g08510)		-2.099
orange1.1t01636	--	Protein indeterminate-domain 14 {ECO:0000303|PubMed:16784536}; AtIDD14; Transcriptional regulator of starch metabolism IDD14 {ECO:0000312|EMBL:AEE34752.1} (At1g68130)	-1.009	
orange1.1t02083	K20617 cytochrome P450 family 71 subfamily A | (RefSeq) cytochrome P450 71A9-like (A)	Cytochrome P450 83B1; EC=1.14.14.45 {ECO:0000269|PubMed:12970475}; Protein ALTERED TRYPTOPHAN REGULATION 4; Protein RED ELONGATED 1; Protein SUPERROOT 2 (At4g31500)		-1.695
orange1.1t02084	K20617 cytochrome P450 family 71 subfamily A | (RefSeq) cytochrome P450 71A9-like (A)	Cytochrome P450 83B1; EC=1.14.14.45 {ECO:0000269|PubMed:12970475}; Protein ALTERED TRYPTOPHAN REGULATION 4; Protein RED ELONGATED 1; Protein SUPERROOT 2 (At4g31500)		-1.790
orange1.1t02796	K20617 cytochrome P450 family 71 subfamily A | (RefSeq) cytochrome P450 71A9-like (A)	Cytochrome P450 83B1; EC=1.14.14.45 {ECO:0000269|PubMed:12970475}; Protein ALTERED TRYPTOPHAN REGULATION 4; Protein RED ELONGATED 1; Protein SUPERROOT 2 (At4g31500)	2.173	-1.863
orange1.1t03558	--	Protein SHI RELATED SEQUENCE 6 (At3g54430)		-1.762
orange1.1t04266	--	Protein indeterminate-domain 14 {ECO:0000303|PubMed:16784536}; AtIDD14; Transcriptional regulator of starch metabolism IDD14 {ECO:0000312|EMBL:AEE34752.1} (At1g68130)	1.303	
Auxin metabolic process [GO:0009850; P = 0.3562 (LN0 vs. LN15) or 0.0552 (RN0 vs. RN15)]				
Cs1g23870	K11816 indole-3-pyruvate monooxygenase [EC:1.14.13.168] | (RefSeq) indole-3-pyruvate monooxygenase YUCCA2-like (A)	Indole-3-pyruvate monooxygenase YUCCA2; EC=1.14.13.168; Flavin-containing monooxygenase YUCCA2 (At4g13260)	1.995	
Cs2g02410	K11147 dehydrogenase/reductase SDR family member 4 [EC:1.1.-.-] | (RefSeq) short-chain dehydrogenase/reductase SDRA (A)	Short-chain dehydrogenase/reductase SDRA {ECO:0000305}; EC=1.1.-.- {ECO:0000305}; Protein INDOLE-3-BUTYRIC ACID RESPONSE 1 {ECO:0000303|PubMed:18725356}; Short-chain dehydrogenase/reductase A {ECO:0000305} (At4g05530)		1.364
Cs2g17930	K01658 anthranilate synthase component II [EC:4.1.3.27] | (RefSeq) anthranilate synthase beta subunit 2, chloroplastic-like (A)	Anthranilate synthase beta subunit 1, chloroplastic; EC=4.1.3.27; Anthranilate synthase component 2-1; Anthranilate synthase, glutamine amidotransferase component 2-1; Protein TRYPTOPHAN BIOSYNTHESIS 4; Protein WEAK ETHYLENE INSENSITIVE 7 (At1g25220)		-1.192

Cs2g18250	K13691 pathogen-inducible salicylic acid glucosyltransferase [EC:2.4.1.-] | (RefSeq) UGT3; UDP-glycosyltransferase 74G1 (A)	UDP-glycosyltransferase 74E2; EC=2.4.1.- (At1g05680)		-1.661
Cs2g23790	K13691 pathogen-inducible salicylic acid glucosyltransferase [EC:2.4.1.-] | (RefSeq) UDP-glycosyltransferase 74E2-like (A)	UDP-glycosyltransferase 74E2; EC=2.4.1.- (At1g05680)		3.046
Cs3g07330	K20617 cytochrome P450 family 71 subfamily A | (RefSeq) cytochrome P450 71A1-like (A)	Cytochrome P450 83B1; EC=1.14.14.45 {ECO:0000269|PubMed:12970475}; Protein ALTERED TRYPTOPHAN REGULATION 4; Protein RED ELONGATED 1; Protein SUPERROOT 2 (At4g31500)	-1.986	
Cs3g25510	--	Protein WALLS ARE THIN 1 (At1g75500)		-1.694
Cs3g25780	K20617 cytochrome P450 family 71 subfamily A | (RefSeq) cytochrome P450 71A9-like (A)	Cytochrome P450 83B1; EC=1.14.14.45 {ECO:0000269|PubMed:12970475}; Protein ALTERED TRYPTOPHAN REGULATION 4; Protein RED ELONGATED 1; Protein SUPERROOT 2 (At4g31500)	2.075	
Cs5g25880	K20617 cytochrome P450 family 71 subfamily A | (RefSeq) cytochrome P450 71A9-like (A)	Cytochrome P450 83B1; EC=1.14.14.45 {ECO:0000269|PubMed:12970475}; Protein ALTERED TRYPTOPHAN REGULATION 4; Protein RED ELONGATED 1; Protein SUPERROOT 2 (At4g31500)		3.612
Cs5g25920	K20617 cytochrome P450 family 71 subfamily A | (RefSeq) cytochrome P450 71A9-like (A)	Cytochrome P450 83B1; EC=1.14.14.45 {ECO:0000269|PubMed:12970475}; Protein ALTERED TRYPTOPHAN REGULATION 4; Protein RED ELONGATED 1; Protein SUPERROOT 2 (At4g31500)		3.960
Cs5g32440	K11816 indole-3-pyruvate monooxygenase [EC:1.14.13.168] | (RefSeq) probable indole-3-pyruvate monooxygenase YUCCA10 (A)	Probable indole-3-pyruvate monooxygenase YUCCA10; EC=1.14.13.168; Flavin-containing monooxygenase YUCCA10 (At1g48910)		-1.757
Cs5g34410	K11816 indole-3-pyruvate monooxygenase [EC:1.14.13.168] | (RefSeq) probable indole-3-pyruvate monooxygenase YUCCA5 (A)	Probable indole-3-pyruvate monooxygenase YUCCA8; EC=1.14.13.168; Flavin-containing monooxygenase YUCCA8 (At4g28720)		-1.767
Cs6g14040	K14664 IAA-amino acid hydrolase [EC:3.5.1.-] | (RefSeq) IAA-amino acid hydrolase ILR1-like 3 (A)	IAA-amino acid hydrolase ILR1 {ECO:0000303|PubMed:7792599}; EC=3.5.1.- {ECO:0000305} (At3g02875)		3.327
Cs7g14760	K05857 phosphatidylinositol phospholipase C, delta [EC:3.1.4.11] | (RefSeq) phosphoinositide phospholipase C 2-like (A)	Phosphoinositide phospholipase C 2; EC=3.1.4.11; Phosphoinositide phospholipase PLC2; AtPLC2; PI-PLC2 (At3g08510)	2.283	
Cs7g22090	--	Protein SHOOT GRAVITROPISM 5; Protein indeterminate-domain 15 {ECO:0000303|PubMed:16784536}; AtIDD15 (At3g48550)		-2.999
Cs7g29470	--	Methylesterase 17; AtMES17; EC=3.1.1.-; Methyl indole-3-acetic acid esterase (At3g10870)		2.337
Cs8g13760	K09842 abscisic-aldehyde oxidase [EC:1.2.3.14] | (RefSeq) AAO3; abscisic-aldehyde oxidase (A)	Indole-3-acetaldehyde oxidase; IAA oxidase; EC=1.2.3.7; Aldehyde oxidase 1; AO-1; AtAO-1; AtAO1 (At5g20960)		2.775
Cs8g13770	K09842 abscisic-aldehyde oxidase [EC:1.2.3.14] | (RefSeq) indole-3-acetaldehyde oxidase-like (A)	Indole-3-acetaldehyde oxidase; IAA oxidase; EC=1.2.3.7; Aldehyde oxidase 1; AO-1; AtAO-1; AtAO1 (At5g20960)		1.065

Cs8g20260	K05857 phosphatidylinositol phospholipase C, delta [EC:3.1.4.11] | (RefSeq) phosphoinositide phospholipase C 2-like (A)	Phosphoinositide phospholipase C 2; EC=3.1.4.11; Phosphoinositide phospholipase PLC2; AtPLC2; PI-PLC2 (At3g08510)		-2.099
orange1.1t01636	--	Protein indeterminate-domain 14 {ECO:0000303|PubMed:16784536}; AtIDD14; Transcriptional regulator of starch metabolism IDD14 {ECO:0000312|EMBL:AEE34752.1} (At1g68130)	-1.009	
orange1.1t02083	K20617 cytochrome P450 family 71 subfamily A | (RefSeq) cytochrome P450 71A9-like (A)	Cytochrome P450 83B1; EC=1.14.14.45 {ECO:0000269|PubMed:12970475}; Protein ALTERED TRYPTOPHAN REGULATION 4; Protein RED ELONGATED 1; Protein SUPERROOT 2 (At4g31500)		-1.695
orange1.1t02084	K20617 cytochrome P450 family 71 subfamily A | (RefSeq) cytochrome P450 71A9-like (A)	Cytochrome P450 83B1; EC=1.14.14.45 {ECO:0000269|PubMed:12970475}; Protein ALTERED TRYPTOPHAN REGULATION 4; Protein RED ELONGATED 1; Protein SUPERROOT 2 (At4g31500)		-1.790
orange1.1t02796	K20617 cytochrome P450 family 71 subfamily A | (RefSeq) cytochrome P450 71A9-like (A)	Cytochrome P450 83B1; EC=1.14.14.45 {ECO:0000269|PubMed:12970475}; Protein ALTERED TRYPTOPHAN REGULATION 4; Protein RED ELONGATED 1; Protein SUPERROOT 2 (At4g31500)	2.173	-1.863
orange1.1t03558	--	Protein SHI RELATED SEQUENCE 6 (At3g54430)		-1.762
orange1.1t04266	--	Protein indeterminate-domain 14 {ECO:0000303|PubMed:16784536}; AtIDD14; Transcriptional regulator of starch metabolism IDD14 {ECO:0000312|EMBL:AEE34752.1} (At1g68130)	1.303	
orange1.1t05657	K13691 pathogen-inducible salicylic acid glucosyltransferase [EC:2.4.1.-] | (RefSeq) UDP-glycosyltransferase 74E2-like (A)	UDP-glycosyltransferase 74E2; EC=2.4.1.- (At1g05680)	3.416	1.776
Cytokinin biosynthetic process [GO:0009691; P = 0.9674 (LN0 vs. LN15) or 0.9332 (RN0 vs. RN15)]				
Cs3g07650	K00791 tRNA dimethylallyltransferase [EC:2.5.1.75] | (RefSeq) tRNA dimethylallyltransferase 2 (A)	tRNA dimethylallyltransferase 2; EC=2.5.1.75; Isopentenyl-diphosphate: tRNA isopentenyltransferase 2; AtIPT2; IPP transferase 2; IPPT 2 (At2g27760)		1.148
Cs3g25930	K06966 uncharacterized protein | (RefSeq) cytokinin riboside 5'-monophosphate phosphoribohydrolase LOG1-like (A)	Cytokinin riboside 5'-monophosphate phosphoribohydrolase LOG1; EC=3.2.2.n1; Protein LONELY GUY 1 (At5g06300)	1.827	
Cs9g17460	K10717 cytokinin trans-hydroxylase | (RefSeq) cytokinin hydroxylase-like (A)	Cytokinin hydroxylase; EC=1.14.13.-; Cytochrome P450 35A1 (At5g52400)		2.111
Cytokinin metabolic process [GO:0009690; P = 0.8054 (LN0 vs. LN15) or 0.8429 (RN0 vs. RN15)]				
Cs3g07650	K00791 tRNA dimethylallyltransferase [EC:2.5.1.75] | (RefSeq) tRNA dimethylallyltransferase 2 (A)	tRNA dimethylallyltransferase 2; EC=2.5.1.75; Isopentenyl-diphosphate: tRNA isopentenyltransferase 2; AtIPT2; IPP transferase 2; IPPT 2 (At2g27760)		1.148
Cs3g25930	K06966 uncharacterized protein | (RefSeq) cytokinin riboside 5'-monophosphate phosphoribohydrolase LOG1-like (A)	Cytokinin riboside 5'-monophosphate phosphoribohydrolase LOG1; EC=3.2.2.n1; Protein LONELY GUY 1 (At5g06300)	1.827	

Cs4g06150	K00279 cytokinin dehydrogenase [EC:1.5.99.12] | (RefSeq) cytokinin dehydrogenase 7 (A)	Cytokinin dehydrogenase 7; EC=1.5.99.12; Cytokinin oxidase 7; AtCKX5; AtCKX7; CKO7 (At2g41510)	-1.275	
Cs4g14450	K00279 cytokinin dehydrogenase [EC:1.5.99.12] | (RefSeq) cytokinin dehydrogenase 3-like (A)	Cytokinin dehydrogenase 3; EC=1.5.99.12; Cytokinin oxidase 3; AtCKX3; CKO 3 (At5g56970)		3.045
Cs4g16640	K13495 cis-zeatin O-glucosyltransferase [EC:2.4.1.215] | (RefSeq) zeatin O-glucosyltransferase-like (A)	Zeatin O-glucosyltransferase; EC=2.4.1.203; Trans-zeatin O-beta-D-glucosyltransferase (At2g15490)		5.084
Cs9g17460	K10717 cytokinin trans-hydroxylase | (RefSeq) cytokinin hydroxylase-like (A)	Cytokinin hydroxylase; EC=1.14.13.-; Cytochrome P450 35A1 (At5g52400)		2.111
orange1.1t00627	K00279 cytokinin dehydrogenase [EC:1.5.99.12] | (RefSeq) cytokinin dehydrogenase 1-like (A)	Cytokinin dehydrogenase 1; EC=1.5.99.12; Cytokinin oxidase 1; AtCKX1 (At2g41510)	-1.392	
orange1.1t02338	K13495 cis-zeatin O-glucosyltransferase [EC:2.4.1.215] | (RefSeq) zeatin O-glucosyltransferase-like (A)	Zeatin O-glucosyltransferase; EC=2.4.1.203; Trans-zeatin O-beta-D-glucosyltransferase (At2g15490)		4.872
Gibberellin biosynthetic process [GO:0009686; P = 0.4608 (LN0 vs. LN15) or 0.0076 (RN0 vs. RN15)]				
Cs5g15530	K04121 ent-kaurene synthase [EC:4.2.3.19] | (RefSeq) ent-kaur-16-ene synthase, chloroplastic (A)	Ent-copalyl diphosphate synthase, chloroplastic; Ent-CDP synthase; EC=5.5.1.13; Ent-copalyl diphosphate synthase; Ent-kaurene synthase A; KSA (At4g02780)	-2.365	
orange1.1t01909	K04122 ent-kaurene oxidase [EC:1.14.13.78] | (RefSeq) ent-kaurene oxidase, chloroplastic-like (A)	Ent-kaurene oxidase, chloroplastic; AtKO1; EC=1.14.13.78 {ECO:0000269|PubMed:20698828, ECO:0000269|PubMed:9952446}; Cytochrome P450 701A3 (At5g25900)	3.520	1.661
orange1.1t01910	K04122 ent-kaurene oxidase [EC:1.14.13.78] | (RefSeq) ent-kaurene oxidase, chloroplastic-like (A)	Ent-kaurene oxidase, chloroplastic; AtKO1; EC=1.14.13.78 {ECO:0000269|PubMed:20698828, ECO:0000269|PubMed:9952446}; Cytochrome P450 701A3 (At5g25900)	1.926	2.918
Cs4g05990	K14510 serine/threonine-protein kinase CTR1 [EC:2.7.11.1] | (RefSeq) serine/threonine-protein kinase CTR1-like (A)	Serine/threonine-protein kinase CTR1 {ECO:0000303|PubMed:8431946}; EC=2.7.11.1 {ECO:0000305}; Protein CONSTITUTIVE TRIPLE RESPONSE1 {ECO:0000303|PubMed:8431946} (At5g03730)		12.570
Cs7g14940	K04125 gibberellin 2-oxidase [EC:1.14.11.13] | (RefSeq) gibberellin 2-beta-dioxygenase 8-like (A)	Gibberellin 2-beta-dioxygenase 8; EC=1.14.11.13; GA 2-oxidase 8; Gibberellin 2-beta-hydroxylase 8; Gibberellin 2-oxidase 8 (At4g21200)		2.657
Cs4g20350	K04124 gibberellin 3-beta-dioxygenase [EC:1.14.11.15] | (RefSeq) gibberellin 3-beta-dioxygenase 1 (A)	Gibberellin 3-beta-dioxygenase 1; EC=1.14.11.15; GA 3-oxidase 1; Gibberellin 3 beta-hydroxylase 1 (At1g15550)		3.048
Cs9g16520	K05282 gibberellin 20-oxidase [EC:1.14.11.12] | (RefSeq) gibberellin 20 oxidase 1 (A)	Gibberellin 20 oxidase 1; EC=1.14.11.-; GA 20-oxidase 1; AtGA20ox1; Gibberellin C-20 oxidase 1 (At4g25420)		2.161
Cs5g31210	K04121 ent-kaurene synthase [EC:4.2.3.19] | (RefSeq) ent-kaur-16-ene synthase, chloroplastic (A)	Ent-copalyl diphosphate synthase, chloroplastic; Ent-CDP synthase; EC=5.5.1.13; Ent-copalyl diphosphate synthase; Ent-kaurene synthase A; KSA (At4g02780)		1.797
orange1.1t03278	K04121 ent-kaurene synthase [EC:4.2.3.19] | (RefSeq)	Ent-kaur-16-ene synthase, chloroplastic; EC=4.2.3.19; Ent-kaurene synthase; AtKS;		2.905

	ent-kaur-16-ene synthase, chloroplastic (A)	Ent-kaurene synthase B; KSB; Protein GA REQUIRING 2 (At1g79460)		
orange1.1t00272	K05282 gibberellin 20-oxidase [EC:1.14.11.12] | (RefSeq) gibberellin 20 oxidase 1-D-like (A)	Gibberellin 20 oxidase 2; EC=1.14.11.-; GA 20-oxidase 2; Gibberellin C-20 oxidase 2 (At5g51810)		7.813
Cs8g20380	K04125 gibberellin 2-oxidase [EC:1.14.11.13] | (RefSeq) gibberellin 2-beta-dioxygenase 8-like (A)	Gibberellin 2-beta-dioxygenase 8; EC=1.14.11.13; GA 2-oxidase 8; Gibberellin 2-beta-hydroxylase 8; Gibberellin 2-oxidase 8 (At5g58660)		-1.272
Cs1g20840	K13422 transcription factor MYC2 | (RefSeq) transcription factor MYC2-like (A)	Transcription factor bHLH93; Basic helix-loop-helix protein 93; AtbHLH93; bHLH 93; Transcription factor EN 47; bHLH transcription factor bHLH093 (At5g65640)		-1.361
Gibberellin metabolic process [GO:0009685; P = 0.4837 (LN0 vs. LN15) or 0.0096 (RN0 vs. RN15)]				
Cs5g15530	K04121 ent-kaurene synthase [EC:4.2.3.19] | (RefSeq) ent-kaur-16-ene synthase, chloroplastic (A)	Ent-copalyl diphosphate synthase, chloroplastic; Ent-CDP synthase; EC=5.5.1.13; Ent-copalyl diphosphate synthase; Ent-kaurene synthase A; KSA (At4g02780)	-2.365	
orange1.1t01909	K04122 ent-kaurene oxidase [EC:1.14.13.78] | (RefSeq) ent-kaurene oxidase, chloroplastic-like (A)	Ent-kaurene oxidase, chloroplastic; AtKO1; EC=1.14.13.78 {ECO:0000269|PubMed:20698828, ECO:0000269|PubMed:9952446}; Cytochrome P450 701A3 (At5g25900)	3.520	1.661
orange1.1t01910	K04122 ent-kaurene oxidase [EC:1.14.13.78] | (RefSeq) ent-kaurene oxidase, chloroplastic-like (A)	Ent-kaurene oxidase, chloroplastic; AtKO1; EC=1.14.13.78 {ECO:0000269|PubMed:20698828, ECO:0000269|PubMed:9952446}; Cytochrome P450 701A3 (At5g25900)	1.926	2.918
Cs4g05990	K14510 serine/threonine-protein kinase CTR1 [EC:2.7.11.1] | (RefSeq) serine/threonine-protein kinase CTR1-like (A)	Serine/threonine-protein kinase CTR1 {ECO:0000303|PubMed:8431946}; EC=2.7.11.1 {ECO:0000305}; Protein CONSTITUTIVE TRIPLE RESPONSE1 {ECO:0000303|PubMed:8431946} (At5g03730)		12.570
Cs7g14940	K04125 gibberellin 2-oxidase [EC:1.14.11.13] | (RefSeq) gibberellin 2-beta-dioxygenase 8-like (A)	Gibberellin 2-beta-dioxygenase 8; EC=1.14.11.13; GA 2-oxidase 8; Gibberellin 2-beta-hydroxylase 8; Gibberellin 2-oxidase 8 (At4g21200)		2.657
Cs4g20350	K04124 gibberellin 3-beta-dioxygenase [EC:1.14.11.15] | (RefSeq) gibberellin 3-beta-dioxygenase 1 (A)	Gibberellin 3-beta-dioxygenase 1; EC=1.14.11.15; GA 3-oxidase 1; Gibberellin 3 beta-hydroxylase 1 (At1g15550)		3.048
Cs9g16520	K05282 gibberellin 20-oxidase [EC:1.14.11.12] | (RefSeq) gibberellin 20 oxidase 1 (A)	Gibberellin 20 oxidase 1; EC=1.14.11.-; GA 20-oxidase 1; AtGA20ox1; Gibberellin C-20 oxidase 1 (At4g25420)		2.161
Cs5g31210	K04121 ent-kaurene synthase [EC:4.2.3.19] | (RefSeq) ent-kaur-16-ene synthase, chloroplastic (A)	Ent-copalyl diphosphate synthase, chloroplastic; Ent-CDP synthase; EC=5.5.1.13; Ent-copalyl diphosphate synthase; Ent-kaurene synthase A; KSA (At4g02780)		1.797
orange1.1t03278	K04121 ent-kaurene synthase [EC:4.2.3.19] | (RefSeq) ent-kaur-16-ene synthase, chloroplastic (A)	Ent-kaur-16-ene synthase, chloroplastic; EC=4.2.3.19; Ent-kaurene synthase; AtKS; Ent-kaurene synthase B; KSB; Protein GA REQUIRING 2 (At1g79460)		2.905
orange1.1t00272	K05282 gibberellin 20-oxidase [EC:1.14.11.12] | (RefSeq) gibberellin 20 oxidase 1-D-like (A)	Gibberellin 20 oxidase 2; EC=1.14.11.-; GA 20-oxidase 2; Gibberellin C-20 oxidase 2 (At5g51810)		7.813
Cs8g20380	K04125 gibberellin 2-oxidase [EC:1.14.11.13] | (RefSeq) gibberellin 2-beta-dioxygenase 8-like (A)	Gibberellin 2-beta-dioxygenase 8; EC=1.14.11.13; GA 2-oxidase 8; Gibberellin 2-beta-hydroxylase 8; Gibberellin 2-oxidase 8 (At5g58660)		-1.272

Cs1g20840	K13422 transcription factor MYC2 | (RefSeq) transcription factor MYC2-like (A)	Transcription factor bHLH93; Basic helix-loop-helix protein 93; AtbHLH93; bHLH 93; Transcription factor EN 47; bHLH transcription factor bHLH093 (At5g65640)		-1.361
Jasmonic acid biosynthetic process [(GO:0009695; P = 0.6680 (LN0 vs. LN15) or 0.0642 (RN0 vs. RN15)]				
Cs3g06080	K10525 allene oxide cyclase [EC:5.3.99.6] | (RefSeq) allene oxide cyclase 4, chloroplastic-like (A)	Allene oxide cyclase, chloroplastic; OsAOC; EC=5.3.99.6; Protein COLEOPTILE PHOTOMORPHOGENESIS 2; Protein HEBIBA (At1g13280)		-3.385
Cs3g27010	K10526 OPC-8:0 CoA ligase 1 [EC:6.2.1.-] | (RefSeq) 4-coumarate--CoA ligase-like 5 (A)	4-coumarate--CoA ligase-like 5; EC=6.2.1.-; 4-coumarate--CoA ligase isoform 9; At4CL9; Peroxisomal OPC-8:0-CoA ligase (At1g20510)	1.370	
Cs5g06980	K01904 4-coumarate--CoA ligase [EC:6.2.1.12] | (RefSeq) 4-coumarate--CoA ligase-like 9 isoform X1 (A)	4-coumarate--CoA ligase-like 9; EC=6.2.1.-; 4-coumarate--CoA ligase isoform 4; At4CL4 (At5g63380)	1.922	
Cs5g06990	K01904 4-coumarate--CoA ligase [EC:6.2.1.12] | (RefSeq) 4-coumarate--CoA ligase-like 9 isoform X1 (A)	4-coumarate--CoA ligase-like 9; EC=6.2.1.-; 4-coumarate--CoA ligase isoform 4; At4CL4 (At5g63380)		1.888
Cs6g18900	K10525 allene oxide cyclase [EC:5.3.99.6] | (RefSeq) allene oxide cyclase 4, chloroplastic (A)	Allene oxide cyclase, chloroplastic; OsAOC; EC=5.3.99.6; Protein COLEOPTILE PHOTOMORPHOGENESIS 2; Protein HEBIBA (At1g13280)		-1.303
Cs6g18910	K10525 allene oxide cyclase [EC:5.3.99.6] | (RefSeq) allene oxide cyclase 4, chloroplastic-like (A)	Allene oxide cyclase, chloroplastic ; OsAOC; EC=5.3.99.6 ; Protein COLEOPTILE PHOTOMORPHOGENESIS 2 ; Protein HEBIBA (At1g13280)		-1.772
Cs7g21790	K10526 OPC-8:0 CoA ligase 1 [EC:6.2.1.-] | (RefSeq) 4-coumarate--CoA ligase-like 5 (A)	4-coumarate--CoA ligase-like 5; EC=6.2.1.-; 4-coumarate--CoA ligase isoform 9; At4CL9; Peroxisomal OPC-8:0-CoA ligase 1 (At1g20510)		6.376
novel.1617	--	Protein GRIM REAPER; Stigma-specific STIG1-like protein GRI; GRIp (At1g53130)		-2.523
novel.2136	K00454 lipoxygenase [EC:1.13.11.12] | (RefSeq) linoleate 13S-lipoxygenase 2-1, chloroplastic (A)	Lipoxygenase 2, chloroplastic; AtLOX2; EC=1.13.11.12 (At1g17420)		-2.987
novel.2517	K10525 allene oxide cyclase [EC:5.3.99.6] | (RefSeq) allene oxide cyclase 4, chloroplastic-like (A)	Allene oxide cyclase, chloroplastic; OsAOC; EC=5.3.99.6; Protein COLEOPTILE PHOTOMORPHOGENESIS 2; Protein HEBIBA (At1g13280)		-2.188
Jasmonic acid metabolic process (GO:0009694; P = 0.7895 (LN0 vs. LN15) or 0.1340 (RN0 vs. RN15)]				
Cs3g06080	K10525 allene oxide cyclase [EC:5.3.99.6] | (RefSeq) allene oxide cyclase 4, chloroplastic-like (A)	Allene oxide cyclase, chloroplastic; OsAOC; EC=5.3.99.6; Protein COLEOPTILE PHOTOMORPHOGENESIS 2; Protein HEBIBA (At1g13280)		-3.385
Cs3g27010	K10526 OPC-8:0 CoA ligase 1 [EC:6.2.1.-] | (RefSeq) 4-coumarate--CoA ligase-like 5 (A)	4-coumarate--CoA ligase-like 5; EC=6.2.1.-; 4-coumarate--CoA ligase isoform 9; At4CL9; Peroxisomal OPC-8:0-CoA ligase 1 (At1g20510)	1.370	
Cs5g06980	K10526 OPC-8:0 CoA ligase 1 [EC:6.2.1.-] | (RefSeq) 4-coumarate--CoA ligase-like 5 (A)	4-coumarate--CoA ligase-like 9; EC=6.2.1.-; 4-coumarate--CoA ligase isoform 4; At4CL4 (At5g63380)	1.922	
Cs5g06990	K01904 4-coumarate--CoA ligase [EC:6.2.1.12] | (RefSeq) 4-coumarate--CoA ligase-like 9 isoform X1 (A)	4-coumarate--CoA ligase-like 9; EC=6.2.1.-; 4-coumarate--CoA ligase isoform 4; At4CL4 (At5g63380)		1.888

Cs6g18900	K10525 allene oxide cyclase [EC:5.3.99.6] | (RefSeq) allene oxide cyclase 4, chloroplastic (A)	Allene oxide cyclase, chloroplastic; OsAOC; EC=5.3.99.6; Protein COLEOPTILE PHOTOMORPHOGENESIS 2; Protein HEBIBA(At1g13280)		-1.303
Cs6g18910	K10525 allene oxide cyclase [EC:5.3.99.6] | (RefSeq) allene oxide cyclase 4, chloroplastic-like (A)	Allene oxide cyclase, chloroplastic ; OsAOC; EC=5.3.99.6 ; Protein COLEOPTILE PHOTOMORPHOGENESIS 2 ; Protein HEBIBA (At1g13280)		-1.772
Cs7g21790	K10526 OPC-8:0 CoA ligase 1 [EC:6.2.1.-] | (RefSeq) 4-coumarate--CoA ligase-like 5 (A)	4-coumarate--CoA ligase-like 5; EC=6.2.1.-; 4-coumarate--CoA ligase isoform 9; At4CL9; Peroxisomal OPC-8:0-CoA ligase 1 (At1g20510)		6.376
novel.1617	--	Protein GRIM REAPER; Stigma-specific STIG1-like protein GRI; GRIp (At1g53130)		-2.523
novel.2136	K00454 lipoxygenase [EC:1.13.11.12] | (RefSeq) linoleate 13S-lipoxygenase 2-1, chloroplastic (A)	Lipoxygenase 2, chloroplastic; AtLOX2; EC=1.13.11.12 (At1g17420)		-2.987
novel.2517	K10525 allene oxide cyclase [EC:5.3.99.6] | (RefSeq) allene oxide cyclase 4, chloroplastic-like (A)	Allene oxide cyclase, chloroplastic; OsAOC; EC=5.3.99.6; Protein COLEOPTILE PHOTOMORPHOGENESIS 2; Protein HEBIBA (At1g13280)		-2.188
Ethylene biosynthetic process [GO:0009693; P = 0.4139 (LN0 vs. LN15) or 0.8228 (RN0 vs. RN15)]				
Cs1g15700	K04124 gibberellin 3-beta-dioxygenase [EC:1.14.11.15] | (RefSeq) gibberellin 3-beta-dioxygenase 1-like (A)	1-aminocyclopropane-1-carboxylate oxidase; ACC oxidase; EC=1.14.17.4; Ethylene-forming enzyme; EFE (At1g79760)		-2.511
Cs3g20140	K05933 aminocyclopropanecarboxylate oxidase [EC:1.14.17.4] | (RefSeq) 1-aminocyclopropane-1-carboxylate oxidase 5 (A)	1-aminocyclopropane-1-carboxylate oxidase 5; ACC oxidase 5; AtACO5; EC=1.14.17.4 (At1g77330)		-1.313
Cs3g16400	K01762 1-aminocyclopropane-1-carboxylate synthase [EC:4.4.1.14] | (RefSeq) 1-aminocyclopropane-1-carboxylate synthase 8 (A)	1-aminocyclopropane-1-carboxylate synthase 8; ACC synthase 8; EC=4.4.1.14; S-adenosyl-L-methionine methylthioadenosine-lyase 8 (At4g37770)		-6.175
Cs5g26130		NAC transcription factor 47 {ECO:0000305}; NAC domain-containing protein 47 {ECO:0000305}; ANAC047 {ECO:0000305}; Protein SPEEDY HYPONASTIC GROWTH {ECO:0000303|PubMed:24363315} (At3g04070)	3.780	
orange1.1t00414	K20772 1-aminocyclopropane-1-carboxylate synthase 1/2/6 [EC:4.4.1.14] | (RefSeq) acs-1, acs2; 1-aminocyclopropane-1-carboxylate synthase 1-like (A)	1-aminocyclopropane-1-carboxylate synthase 2; ACC synthase 2; EC=4.4.1.14; S-adenosyl-L-methionine methylthioadenosine-lyase 2 (At1g01480)	1.719	
Cs2g20590	K05933 aminocyclopropanecarboxylate oxidase [EC:1.14.17.4] | (RefSeq) aco-1; ACC oxidase (A)	1-aminocyclopropane-1-carboxylate oxidase; ACC oxidase; EC=1.14.17.4; Ethylene-forming enzyme; EFE (At1g05010)	1.709	
Ethylene metabolic process [GO:0009692; P = 0.4139 (LN0 vs. LN15) or 0.8228 (RN0 vs. RN15)]				
Cs1g15700	K04124 gibberellin 3-beta-dioxygenase [EC:1.14.11.15] | (RefSeq) gibberellin 3-beta-dioxygenase 1-like (A)	1-aminocyclopropane-1-carboxylate oxidase; ACC oxidase; EC=1.14.17.4; Ethylene-forming enzyme; EFE (At1g79760)		-2.511
Cs3g20140	K05933 aminocyclopropanecarboxylate oxidase [EC:1.14.17.4] | (RefSeq) 1-aminocyclopropane-1-carboxylate oxidase 5 (A)	1-aminocyclopropane-1-carboxylate oxidase 5; ACC oxidase 5; AtACO5; EC=1.14.17.4 (At1g77330)		-1.313

Cs3g16400	K01762 1-aminocyclopropane-1-carboxylate synthase [EC:4.4.1.14] | (RefSeq) 1-aminocyclopropane-1-carboxylate synthase 8 (A)	1-aminocyclopropane-1-carboxylate synthase 8; ACC synthase 8; EC=4.4.1.14; S-adenosyl-L-methionine methylthioadenosine-lyase 8 (At4g37770)		-6.175
Cs5g26130		NAC transcription factor 47 {ECO:0000305}; NAC domain-containing protein 47 {ECO:0000305}; ANAC047 {ECO:0000305}; Protein SPEEDY HYPONASTIC GROWTH {ECO:0000303|PubMed:24363315} (At3g04070)	3.780	
orange1.1t00414	K20772 1-aminocyclopropane-1-carboxylate synthase 1/2/6 [EC:4.4.1.14] | (RefSeq) acs-1, acs2; 1-aminocyclopropane-1-carboxylate synthase 1-like (A)	1-aminocyclopropane-1-carboxylate synthase 2; ACC synthase 2; EC=4.4.1.14; S-adenosyl-L-methionine methylthioadenosine-lyase 2 (At1g01480)	1.719	
Cs2g20590	K05933 aminocyclopropanecarboxylate oxidase [EC:1.14.17.4] | (RefSeq) aco-1; ACC oxidase (A)	1-aminocyclopropane-1-carboxylate oxidase; ACC oxidase; EC=1.14.17.4; Ethylene-forming enzyme; EFE (At1g05010)	1.709	
Abscisic acid biosynthetic process [GO:0009688; P = 0.4608 (LN0 vs. LN15) or 0.1046 (RN0 vs. RN15)]				
Cs8g13760	K09842 abscisic-aldehyde oxidase [EC:1.2.3.14] | (RefSeq) AAO3; abscisic-aldehyde oxidase (A)	Indole-3-acetaldehyde oxidase; IAA oxidase; EC=1.2.3.7; Aldehyde oxidase 1; AO-1; AtAO-1; AtAO1 (At5g20960)		2.775
orange1.1t04051	K09838 zeaxanthin epoxidase [EC:1.14.15.21] | (RefSeq) zeaxanthin epoxidase, chloroplastic-like (A)	Zeaxanthin epoxidase, chloroplastic; EC=1.14.15.21; PA-ZE (At5g67030)		6.739
Cs4g12520	K15631 molybdenum cofactor sulfurtransferase [EC:2.8.1.9] | (RefSeq) ABA3; molybdenum cofactor sulfurase (A)	Molybdenum cofactor sulfurase {ECO:0000255|HAMAP-Rule:MF_03050}; MCS {ECO:0000255|HAMAP-Rule:MF_03050}; MOS {ECO:0000255|HAMAP-Rule:MF_03050}; MoCo sulfurase {ECO:0000255|HAMAP-Rule:MF_03050}; EC=2.8.1.9 {ECO:0000255|HAMAP-Rule:MF_03050, ECO:0000269|PubMed:11553608, ECO:0000269|PubMed:15561708}; Abscisic acid protein 3; Low expression of osmotically expressive genes protein 5; Molybdenum cofactor sulfurtransferase {ECO:0000255|HAMAP-Rule:MF_03050} (At1g16540)		1.756
Cs8g13770	K09842 abscisic-aldehyde oxidase [EC:1.2.3.14] | (RefSeq) indole-3-acetaldehyde oxidase-like (A)	Indole-3-acetaldehyde oxidase; IAA oxidase; EC=1.2.3.7; Aldehyde oxidase 1; AO-1; AtAO-1; AtAO1 (At5g20960)		1.065
orange1.1t05125	K09838 zeaxanthin epoxidase [EC:1.14.15.21] | (RefSeq) CitZEP, ZEP; zeaxanthin epoxidase, chloroplastic (A)	Zeaxanthin epoxidase, chloroplastic; EC=1.14.15.21 (At5g11330)		2.122
orange1.1t04849	K09838 zeaxanthin epoxidase [EC:1.14.15.21] | (RefSeq) CitZEP, ZEP; zeaxanthin epoxidase, chloroplastic (A)	Zeaxanthin epoxidase, chloroplastic; AtZEP; EC=1.14.15.21; Protein ABA DEFICIENT 1; AtABA1; Protein IMPAIRED IN BABA-INDUCED STERILITY 3; Protein LOW EXPRESSION OF OSMOTIC STRESS-RESPONSIVE GENES 6; Protein NON-PHOTOCHEMICAL QUENCHING 2 (At5g11330)		2.419
novel.774		NDR1/HIN1-like protein 6 {ECO:0000303|PubMed:26849212} (At5g11890)		-1.783
Cs1g22620	K09838 zeaxanthin epoxidase [EC:1.14.15.21] | (RefSeq) CitZEP,	Zeaxanthin epoxidase, chloroplastic; EC=1.14.15.21; PA-ZE (At5g67030)		-1.027

	ZEP; zeaxanthin epoxidase, chloroplastic (A)			
Cs4g20590	K09838 zeaxanthin epoxidase [EC:1.14.15.21] | (RefSeq) zeaxanthin epoxidase, chloroplastic-like (A)	Zeaxanthin epoxidase, chloroplastic; EC=1.14.15.21 (At5g11330)	-1.719	
Cs2g03270	K09840 9-cis-epoxycarotenoid dioxygenase [EC:1.13.11.51] | (RefSeq) CitNCED2; 9-cis-epoxycarotenoid dioxygenase 2 (A)	9-cis-epoxycarotenoid dioxygenase NCED1, chloroplastic; EC=1.13.11.51; PvNCED1 (At1g30100)	-5.357	
Cs4g20560	K09838 zeaxanthin epoxidase [EC:1.14.15.21] | (RefSeq) zeaxanthin epoxidase, chloroplastic-like (A)	Zeaxanthin epoxidase, chloroplastic; EC=1.14.15.21 (At5g11330)	-1.299	
Abscisic acid metabolic process [GO:0009687; P = 0.6282 (LN0 vs. LN15) or 0.0374 (RN0 vs. RN15)]				
Cs3g21210	K09843 (+)-abscisic acid 8'-hydroxylase [EC:1.14.13.93] | (RefSeq) CYP707A2; abscisic acid 8'-hydroxylase 2-like (A)	Abscisic acid 8'-hydroxylase 3; ABA 8'-hydroxylase 3; EC=1.14.13.93; Cytochrome P450 707A3 (At5g45340)		8.454
Cs1g24480	K04123 ent-kaurenoic acid hydroxylase [EC:1.14.13.79] | (RefSeq) ent-kaurenoic acid oxidase 2-like (A)	Abscisic acid 8'-hydroxylase 3; ABA 8'-hydroxylase 3; EC=1.14.13.93; Cytochrome P450 707A7; OsABA8ox3 (At1g19630)		4.003
Cs8g13760	K09842 abscisic-aldehyde oxidase [EC:1.2.3.14] | (RefSeq) AAO3; abscisic-aldehyde oxidase (A)	Indole-3-acetaldehyde oxidase; IAA oxidase; EC=1.2.3.7; Aldehyde oxidase 1; AO-1; AtAO-1; AtAO1 (At5g20960)		2.775
orange1.1t04051	K09838 zeaxanthin epoxidase [EC:1.14.15.21] | (RefSeq) zeaxanthin epoxidase, chloroplastic-like (A)	Zeaxanthin epoxidase, chloroplastic; EC=1.14.15.21; PA-ZE (At5g67030)		6.739
Cs4g12520	K15631 molybdenum cofactor sulfurtransferase [EC:2.8.1.9] | (RefSeq) ABA3; molybdenum cofactor sulfurase (A)	Molybdenum cofactor sulfurase {ECO:0000255|HAMAP-Rule:MF_03050}; MCS {ECO:0000255|HAMAP-Rule:MF_03050}; MOS {ECO:0000255|HAMAP-Rule:MF_03050}; MoCo sulfurase {ECO:0000255|HAMAP-Rule:MF_03050}; EC=2.8.1.9 {ECO:0000255|HAMAP-Rule:MF_03050, ECO:0000269|PubMed:11553608, ECO:0000269|PubMed:15561708}; Abscisic acid protein 3; Low expression of osmotically expressive genes protein 5; Molybdenum cofactor sulfurtransferase {ECO:0000255|HAMAP-Rule:MF_03050} (At1g16540)		1.756
novel.774		NDR1/HIN1-like protein 6 {ECO:0000303|PubMed:26849212} (At5g11890)		-1.783
Cs8g13770	K09842 abscisic-aldehyde oxidase [EC:1.2.3.14] | (RefSeq) indole-3-acetaldehyde oxidase-like (A)	Indole-3-acetaldehyde oxidase; IAA oxidase; EC=1.2.3.7; Aldehyde oxidase 1; AO-1; AtAO-1; AtAO1 (At5g20960)		1.065
Cs1g22620	K09838 zeaxanthin epoxidase [EC:1.14.15.21] | (RefSeq) CitZEP, ZEP; zeaxanthin epoxidase, chloroplastic (A)	Zeaxanthin epoxidase, chloroplastic; EC=1.14.15.21; PA-ZE (At5g67030)		-1.027
orange1.1t05125	K09838 zeaxanthin epoxidase [EC:1.14.15.21] | (RefSeq) CitZEP, ZEP; zeaxanthin epoxidase, chloroplastic (A)	Zeaxanthin epoxidase, chloroplastic; EC=1.14.15.21 (At5g11330)		2.122
orange1.1t04849	K09838 zeaxanthin epoxidase [EC:1.14.15.21] | (RefSeq) CitZEP, ZEP; zeaxanthin epoxidase, chloroplastic (A)	Zeaxanthin epoxidase, chloroplastic; AtZEP; EC=1.14.15.21; Protein ABA DEFICIENT 1; AtABA1; Protein IMPAIRED IN BABA-INDUCED STERILITY 3;		2.419

		Protein LOW EXPRESSION OF OSMOTIC STRESS-RESPONSIVE GENES 6; Protein NON-PHOTOCHEMICAL QUENCHING 2 (At5g11330)		
Cs3g23530	K09843 (+)-abscisic acid 8'-hydroxylase [EC:1.14.13.93] | (RefSeq) CYP707A4; abscisic acid 8'-hydroxylase 3-like (A)	Abscisic acid 8'-hydroxylase 3; ABA 8'-hydroxylase 3; EC=1.14.13.93; Cytochrome P450 707A7; OsABA8ox3 (At3g19270)		-2.327
Cs4g20590	K09838 zeaxanthin epoxidase [EC:1.14.15.21] | (RefSeq) zeaxanthin epoxidase, chloroplastic-like (A)	Zeaxanthin epoxidase, chloroplastic; EC=1.14.15.21 (At5g11330)	-1.719	
Cs2g03270	K09840 9-cis-epoxycarotenoid dioxygenase [EC:1.13.11.51] | (RefSeq) CitNCED2; 9-cis-epoxycarotenoid dioxygenase 2 (A)	9-cis-epoxycarotenoid dioxygenase NCED1, chloroplastic; EC=1.13.11.51; PvNCED1 (At1g30100)	-5.357	
Cs4g20560	K09838 zeaxanthin epoxidase [EC:1.14.15.21] | (RefSeq) zeaxanthin epoxidase, chloroplastic-like (A)	Zeaxanthin epoxidase, chloroplastic; EC=1.14.15.21 (At5g11330)	-1.299	
DEGs related to strigolactone (SL) biosynthesis and degradation				
Cs4g19460	K17913 carlactone synthase / all-trans-10'-apo-beta-carotenal 13,14-cleaving dioxygenase [EC:1.13.11.69 1.13.11.70] | (RefSeq) carotenoid cleavage dioxygenase 8 homolog B, chloroplastic-like (A)	Carotenoid cleavage dioxygenase 8 homolog B, chloroplastic; OsCCD8b; EC=1.13.11.69 {ECO:0000269|PubMed:22422982}; EC=1.13.11.70 {ECO:0000269|PubMed:18637791}; Protein DWARF-10; Protein MAX4 homolog (At4g32810)		6.102
Cs5g30540	K17911 beta-carotene isomerase [EC:5.2.1.14] | (RefSeq) beta-carotene isomerase D27, chloroplastic (A)	Beta-carotene isomerase D27, chloroplastic; EC=5.2.1.14; Protein DWARF-27 (At1g64680)		5.919
Cs1g25090	K17912 9-cis-beta-carotene 9',10'-cleaving dioxygenase [EC:1.13.11.68] | (RefSeq) carotenoid cleavage dioxygenase 7, chloroplastic (A)	Carotenoid cleavage dioxygenase 7, chloroplastic; AtCCD7; AtNCED7; Beta,beta-carotene 9',10'-oxygenase; EC=1.13.11.68; Protein MORE AXILLARY BRANCHING 3; Protein MORE AXILLARY GROWTH 3 (At2g44990)		7.476
Cs4g16860	K13699 abhydrolase domain-containing protein 5 [EC:2.3.1.51] | (RefSeq) 1-acylglycerol-3-phosphate O-acyltransferase (A)	Probable strigolactone esterase DAD2; 3.1.-.-; Protein DECREASED APICAL DOMINANCE 2 (At3g24420)		3.907
Cs4g19470	K17913 carlactone synthase / all-trans-10'-apo-beta-carotenal 13,14-cleaving dioxygenase [EC:1.13.11.69 1.13.11.70] | (RefSeq) carotenoid cleavage dioxygenase 8 homolog B, chloroplastic-like (A)	Carotenoid cleavage dioxygenase 8 homolog B, chloroplastic; OsCCD8b; EC=1.13.11.69 {ECO:0000269|PubMed:22422982}; EC=1.13.11.70 {ECO:0000269|PubMed:18637791}; Protein DWARF-10; Protein MAX4 homolog (At4g32810)		-1.097
Cs3g16030		Strigolactone esterase D14 {ECO:0000303|PubMed:25425668}; EC=3.1.-.- {ECO:0000269|PubMed:25425668}; Protein DWARF 14 {ECO:0000303|PubMed:22357928}; AtD14 {ECO:0000303|PubMed:22357928} (At3g03990)		-1.244
Salicylic acid biosynthetic process [GO:0009697; P = 0.8274 (RN0 vs. RN15roots)]				
novel.1617	--	Protein GRIM REAPER; Stigma-specific STIG1-like protein GRI; GRIp		-2.523

		(At1g53130)		
Salicylic acid catabolic process [GO:0046244; P = 0.0208 (leaves) or 0.0500 (RN0 vs RN15)]				
Cs1g12310	K24028 salicylic acid 3-hydroxylase [EC:1.14.11.-] | (RefSeq) protein DMR6-LIKE OXYGENASE 2-like (A)	Protein DMR6-LIKE OXYGENASE 1 {ECO:0000303|PubMed:25376907}; EC=1.14.11.- {ECO:0000255|PROSITE-ProRule:PRU00805}; 2-oxoglutarate (2OG)-Fe(II) oxygenase-like protein DLO1 {ECO:0000303|PubMed:25376907}; Protein SENESCENCE-ASSOCIATED GENE 108 {ECO:0000303|PubMed:23959884}; Salicylate 3-hydroxylase DLO1 {ECO:0000303|PubMed:23959884}; S3H DLO1 {ECO:0000303|PubMed:23959884}; SA 3-hydroxylase DLO1 {ECO:0000303|PubMed:23959884}; Salicylic acid 3-hydroxylase DLO1 {ECO:0000303|PubMed:23959884}; EC=1.14.13.- {ECO:0000269|PubMed:23959884} (At4g10500)		-1.942
Cs5g16310	K24028 salicylic acid 3-hydroxylase [EC:1.14.11.-] | (RefSeq) protein DOWNY MILDEW RESISTANCE 6 (A)	Protein DOWNY MILDEW RESISTANCE 6 {ECO:0000303|PubMed:15986928}; AtDMR6 {ECO:0000303|PubMed:15986928}; EC=1.14.11.- {ECO:0000255|PROSITE-ProRule:PRU00805};2-oxoglutarate (2OG)-Fe(II) oxygenase-like protein DMR6 {ECO:0000303|PubMed:18248595};Salicylate 3-hydroxylase DMR6 {ECO:0000305}; S3H DMR6 {ECO:0000305}; SA 3-hydroxylase DMR6 {ECO:0000305}; Salicylic acid 3-hydroxylase DMR6 {ECO:0000305}; EC=1.14.13.- {ECO:0000250|UniProtKB:Q9ZSA8} (At5g24530)	1.864	
Cs5g28710	K24028 salicylic acid 3-hydroxylase [EC:1.14.11.-] | (RefSeq) protein DMR6-LIKE OXYGENASE 2-like (A)	Protein DMR6-LIKE OXYGENASE 1 {ECO:0000303|PubMed:25376907}; EC=1.14.11.- {ECO:0000255|PROSITE-ProRule:PRU00805}; A2-oxoglutarate (2OG)-Fe(II) oxygenase-like protein DLO1 {ECO:0000303|PubMed:25376907}; Protein SENESCENCE-ASSOCIATED GENE 108 {ECO:0000303|PubMed:23959884}; Salicylate 3-hydroxylase DLO1 {ECO:0000303|PubMed:23959884}; S3H DLO1 {ECO:0000303|PubMed:23959884}; SA 3-hydroxylase DLO1 {ECO:0000303|PubMed:23959884}; Salicylic acid 3-hydroxylase DLO1 {ECO:0000303|PubMed:23959884}; EC=1.14.13.- {ECO:0000269|PubMed:23959884} (At3g19000)		-2.993
Cs5g28720	--	Protein DMR6-LIKE OXYGENASE 1 {ECO:0000303|PubMed:25376907}; EC=1.14.11.- {ECO:0000255|PROSITE-ProRule:PRU00805};2-oxoglutarate (2OG)-Fe(II) oxygenase-like protein DLO1 {ECO:0000303|PubMed:25376907}; Protein SENESCENCE-ASSOCIATED GENE 108		4.401

		{ECO:0000303|PubMed:23959884}; Salicylate 3-hydroxylase DLO1 {ECO:0000303|PubMed:23959884}; S3H DLO1 {ECO:0000303|PubMed:23959884}; SA 3-hydroxylase DLO1 {ECO:0000303|PubMed:23959884}; Salicylic acid 3-hydroxylase DLO1 {ECO:0000303|PubMed:23959884}; EC=1.14.13.- {ECO:0000269|PubMed:23959884} (At3g19000)		
Cs5g28730	K24028 salicylic acid 3-hydroxylase [EC:1.14.11.-] | (RefSeq) protein DMR6-LIKE OXYGENASE 2-like (A)	Protein DMR6-LIKE OXYGENASE 1 {ECO:0000303|PubMed:25376907}; EC=1.14.11.- {ECO:0000255|PROSITE-ProRule:PRU00805}; 2-oxoglutarate (2OG)-Fe(II) oxygenase-like protein DLO1 {ECO:0000303|PubMed:25376907}; Protein SENESCENCE-ASSOCIATED GENE 108 {ECO:0000303|PubMed:23959884};Salicylate 3-hydroxylase DLO1 {ECO:0000303|PubMed:23959884}; S3H DLO1 {ECO:0000303|PubMed:23959884}; SA 3-hydroxylase DLO1 {ECO:0000303|PubMed:23959884}; Salicylic acid 3-hydroxylase DLO1 {ECO:0000303|PubMed:23959884}; EC=1.14.13.- {ECO:0000269|PubMed:23959884} (At3g19000)	-2.437	
Cs5g28750	K24028 salicylic acid 3-hydroxylase [EC:1.14.11.-] | (RefSeq) protein DOWNY MILDEW RESISTANCE 6 (A)	Protein DMR6-LIKE OXYGENASE 1 {ECO:0000303|PubMed:25376907}; EC=1.14.11.- {ECO:0000255|PROSITE-ProRule:PRU00805}; 2-oxoglutarate (2OG)-Fe(II) oxygenase-like protein DLO1 {ECO:0000303|PubMed:25376907}; Protein SENESCENCE-ASSOCIATED GENE 108 {ECO:0000303|PubMed:23959884}; Salicylate 3-hydroxylase DLO1 {ECO:0000303|PubMed:23959884}; S3H DLO1 {ECO:0000303|PubMed:23959884}; SA 3-hydroxylase DLO1 {ECO:0000303|PubMed:23959884}; Salicylic acid 3-hydroxylase DLO1 {ECO:0000303|PubMed:23959884}; EC=1.14.13.- {ECO:0000269|PubMed:23959884} (At3g19000)		4.442
Cs5g28780	K24028 salicylic acid 3-hydroxylase [EC:1.14.11.-] | (RefSeq) protein DMR6-LIKE OXYGENASE 2-like (A)	Protein DMR6-LIKE OXYGENASE 2 {ECO:0000303|PubMed:25376907}; EC=1.14.11.- {ECO:0000255|PROSITE-ProRule:PRU00805}; 2-oxoglutarate (2OG)-Fe(II) oxygenase-like protein DLO2 {ECO:0000303|PubMed:25376907}; Salicylate 3-hydroxylase DLO2 {ECO:0000305}; S3H DLO2 {ECO:0000305}; SA 3-hydroxylase DLO2 {ECO:0000305}; Salicylic acid 3-hydroxylase DLO2 {ECO:0000305}; EC=1.14.13.- {ECO:0000250|UniProtKB:Q9ZSA8} (At3g19000)		-1.274
Cs9g14480	K24028 salicylic acid 3-hydroxylase [EC:1.14.11.-] | (RefSeq) protein DMR6-LIKE OXYGENASE 2-like (A)	Protein DMR6-LIKE OXYGENASE 2 {ECO:0000303|PubMed:25376907}; EC=1.14.11.- {ECO:0000255|PROSITE-ProRule:PRU00805}; 2-oxoglutarate (2OG)-Fe(II) oxygenase-like protein DLO2 {ECO:0000303|PubMed:25376907};	6.705	

		Salicylate 3-hydroxylase DLO2 {ECO:0000305}; S3H DLO2 {ECO:0000305}; SA 3-hydroxylase DLO2 {ECO:0000305}; Salicylic acid 3-hydroxylase DLO2 {ECO:0000305}; EC=1.14.13.- {ECO:0000250|UniProtKB:Q9ZSA8} (At4g10490)		
Cs9g14500	K24028 salicylic acid 3-hydroxylase [EC:1.14.11.-] | (RefSeq) protein DMR6-LIKE OXYGENASE 2-like (A)	Protein DMR6-LIKE OXYGENASE 2 {ECO:0000303|PubMed:25376907}; EC=1.14.11.- {ECO:0000255|PROSITE-ProRule:PRU00805}; 2-oxoglutarate (2OG)-Fe(II) oxygenase-like protein DLO2 {ECO:0000303|PubMed:25376907}; Salicylate 3-hydroxylase DLO2 {ECO:0000305}; S3H DLO2 {ECO:0000305}; SA 3-hydroxylase DLO2 {ECO:0000305}; Salicylic acid 3-hydroxylase DLO2 {ECO:0000305}; EC=1.14.13.- {ECO:0000250|UniProtKB:Q9ZSA8} (At4g10490)		1.351
orange1.1t01963	K24028 salicylic acid 3-hydroxylase [EC:1.14.11.-] | (RefSeq) protein DMR6-LIKE OXYGENASE 2-like (A)	Protein DMR6-LIKE OXYGENASE 2 {ECO:0000303|PubMed:25376907}; EC=1.14.11.- {ECO:0000255|PROSITE-ProRule:PRU00805}; 2-oxoglutarate (2OG)-Fe(II) oxygenase-like protein DLO2 {ECO:0000303|PubMed:25376907}; Salicylate 3-hydroxylase DLO2 {ECO:0000305}; S3H DLO2 {ECO:0000305}; SA 3-hydroxylase DLO2 {ECO:0000305};Salicylic acid 3-hydroxylase DLO2 {ECO:0000305}; EC=1.14.13.- {ECO:0000250|UniProtKB:Q9ZSA8} (At4g10490)	1.425	
Salicylic acid metabolic process [GO:0009696; P = 0.0006 (LN0 vs. LN15) or 0.0188 (RN0 vs. RN15)]				
Cs1g08280	K18875 enhanced disease susceptibility 1 protein | (RefSeq) protein EDS1-like (A)	Lipase-like PAD4; EC=2.3.1.-; Protein ENHANCED DISEASE SUSCEPTIBILITY 9; Protein PHYTOALEXIN DEFICIENT 4; AtPAD4 (At3g52430)	2.042	
Cs1g12310	K24028 salicylic acid 3-hydroxylase [EC:1.14.11.-] | (RefSeq) protein DMR6-LIKE OXYGENASE 2-like (A)	Protein DMR6-LIKE OXYGENASE 1 {ECO:0000303|PubMed:25376907}; EC=1.14.11.- {ECO:0000255|PROSITE-ProRule:PRU00805}; 2-oxoglutarate (2OG)-Fe(II) oxygenase-like protein DLO1 {ECO:0000303|PubMed:25376907}; Protein SENESCENCE-ASSOCIATED GENE 108 {ECO:0000303|PubMed:23959884}; Salicylate 3-hydroxylase DLO1 {ECO:0000303|PubMed:23959884}; S3H DLO1 {ECO:0000303|PubMed:23959884}; SA 3-hydroxylase DLO1 {ECO:0000303|PubMed:23959884}; Salicylic acid 3-hydroxylase DLO1 {ECO:0000303|PubMed:23959884}; EC=1.14.13.- {ECO:0000269|PubMed:23959884} (At4g10500)		-1.942
Cs2g18240	K13691 pathogen-inducible salicylic acid glucosyltransferase [EC:2.4.1.-] | (RefSeq) UDP-glycosyltransferase 74G1-like (A)	UDP-glycosyltransferase 74F2; EC=2.4.1.-; AtSGT1; Salicylic acid glucosyltransferase 1 (At2g43820)		-1.269
Cs2g18300	K13691 pathogen-inducible salicylic acid glucosyltransferase [EC:2.4.1.-] | (RefSeq) UDP-glycosyltransferase 74E2-like (A)	UDP-glycosyltransferase 74F2; EC=2.4.1.-; AtSGT1; Salicylic acid glucosyltransferase 1 (At2g43820)		1.921
Cs5g16310	K24028 salicylic acid 3-hydroxylase [EC:1.14.11.-] | (RefSeq)	Protein DOWNY MILDEW RESISTANCE 6 {ECO:0000303|PubMed:15986928};	1.864	

	protein DOWNY MILDEW RESISTANCE 6 (A)	AtDMR6 {ECO:0000303|PubMed:15986928}; EC=1.14.11.- {ECO:0000255|PROSITE-ProRule:PRU00805};2-oxoglutarate (2OG)-Fe(II) oxygenase-like protein DMR6 {ECO:0000303|PubMed:18248595};Salicylate 3-hydroxylase DMR6 {ECO:0000305}; S3H DMR6 {ECO:0000305}; SA 3-hydroxylase DMR6 {ECO:0000305}; Salicylic acid 3-hydroxylase DMR6 {ECO:0000305}; EC=1.14.13.- {ECO:0000250|UniProtKB:Q9ZSA8} (At5g24530)		
Cs5g18300	K01381 saccharopepsin [EC:3.4.23.25] | (RefSeq) aspartic proteinase CDR1-like (A)	Aspartic proteinase CDR1; EC=3.4.23.-; Protein CONSTITUTIVE DISEASE RESISTANCE 1 (At1g64830)	2.519	-2.890
Cs5g18330	K01381 saccharopepsin [EC:3.4.23.25] | (RefSeq) aspartic proteinase CDR1-like (A)	Aspartic proteinase CDR1; EC=3.4.23.-; Protein CONSTITUTIVE DISEASE RESISTANCE 1 (At1g64830)		-7.269
Cs5g21200	K13691 pathogen-inducible salicylic acid glucosyltransferase [EC:2.4.1.-] | (RefSeq) UDP-glycosyltransferase 74F2-like (A)	UDP-glycosyltransferase 74F2; EC=2.4.1.-; AtSGT1; Salicylic acid glucosyltransferase 1; (At2g43820)	1.210	-1.666
Cs5g21220	K13691 pathogen-inducible salicylic acid glucosyltransferase [EC:2.4.1.-] | (RefSeq) UDP-glycosyltransferase 74F2-like (A)	UDP-glycosyltransferase 74F2; EC=2.4.1.-; AtSGT1; Salicylic acid glucosyltransferase 1 (At2g43820)	1.399	-2.142
Cs5g24680	K20547 basic endochitinase B [EC:3.2.1.14] | (RefSeq) basic endochitinase (A)	Chitinase-like protein 1; AtCTL1; Protein ANION ALTERED ROOT MORPHOLOGY; Protein ECTOPIC DEPOSITION OF LIGNIN IN PITH 1; Protein ECTOPIC ROOT HAIR 2; Protein POM-POM1; Protein SENSITIVE TO HOT TEMPERATURES 2 (At1g05850)	-1.216	-1.617
Cs5g28710	K24028 salicylic acid 3-hydroxylase [EC:1.14.11.-] | (RefSeq) protein DMR6-LIKE OXYGENASE 2-like (A)	Protein DMR6-LIKE OXYGENASE 1 {ECO:0000303|PubMed:25376907}; EC=1.14.11.- {ECO:0000255|PROSITE-ProRule:PRU00805}; A2-oxoglutarate (2OG)-Fe(II) oxygenase-like protein DLO1 {ECO:0000303|PubMed:25376907}; Protein SENESCENCE-ASSOCIATED GENE 108 {ECO:0000303|PubMed:23959884}; Salicylate 3-hydroxylase DLO1 {ECO:0000303|PubMed:23959884}; S3H DLO1 {ECO:0000303|PubMed:23959884}; SA 3-hydroxylase DLO1 {ECO:0000303|PubMed:23959884}; Salicylic acid 3-hydroxylase DLO1 {ECO:0000303|PubMed:23959884}; EC=1.14.13.- {ECO:0000269|PubMed:23959884} (At3g19000)		-2.993
Cs5g28720	--	Protein DMR6-LIKE OXYGENASE 1 {ECO:0000303|PubMed:25376907}; EC=1.14.11.- {ECO:0000255|PROSITE-ProRule:PRU00805};2-oxoglutarate (2OG)-Fe(II) oxygenase-like protein DLO1 {ECO:0000303|PubMed:25376907}; Protein SENESCENCE-ASSOCIATED GENE 108 {ECO:0000303|PubMed:23959884}; Salicylate 3-hydroxylase DLO1 {ECO:0000303|PubMed:23959884}; S3H DLO1 {ECO:0000303|PubMed:23959884}; SA 3-hydroxylase DLO1		4.401

		{ECO:0000303|PubMed:23959884}; Salicylic acid 3-hydroxylase DLO1 {ECO:0000303|PubMed:23959884}; EC=1.14.13.- {ECO:0000269|PubMed:23959884} (At3g19000)		
Cs5g28730	K24028 salicylic acid 3-hydroxylase [EC:1.14.11.-] | (RefSeq) protein DMR6-LIKE OXYGENASE 2-like (A)	Protein DMR6-LIKE OXYGENASE 1 {ECO:0000303|PubMed:25376907}; EC=1.14.11.- {ECO:0000255|PROSITE-ProRule:PRU00805}; 2-oxoglutarate (2OG)-Fe(II) oxygenase-like protein DLO1 {ECO:0000303|PubMed:25376907}; Protein SENESCENCE-ASSOCIATED GENE 108 {ECO:0000303|PubMed:23959884};Salicylate 3-hydroxylase DLO1 {ECO:0000303|PubMed:23959884}; S3H DLO1 {ECO:0000303|PubMed:23959884}; SA 3-hydroxylase DLO1 {ECO:0000303|PubMed:23959884}; Salicylic acid 3-hydroxylase DLO1 {ECO:0000303|PubMed:23959884}; EC=1.14.13.- {ECO:0000269|PubMed:23959884} (At3g19000)	-2.437	
Cs5g28750	K24028 salicylic acid 3-hydroxylase [EC:1.14.11.-] | (RefSeq) protein DOWNY MILDEW RESISTANCE 6 (A)	Protein DMR6-LIKE OXYGENASE 1 {ECO:0000303|PubMed:25376907}; EC=1.14.11.- {ECO:0000255|PROSITE-ProRule:PRU00805}; 2-oxoglutarate (2OG)-Fe(II) oxygenase-like protein DLO1 {ECO:0000303|PubMed:25376907}; Protein SENESCENCE-ASSOCIATED GENE 108 {ECO:0000303|PubMed:23959884}; Salicylate 3-hydroxylase DLO1 {ECO:0000303|PubMed:23959884}; S3H DLO1 {ECO:0000303|PubMed:23959884}; SA 3-hydroxylase DLO1 {ECO:0000303|PubMed:23959884}; Salicylic acid 3-hydroxylase DLO1 {ECO:0000303|PubMed:23959884}; EC=1.14.13.- {ECO:0000269|PubMed:23959884} (At3g19000)		4.442
Cs5g28780	K24028 salicylic acid 3-hydroxylase [EC:1.14.11.-] | (RefSeq) protein DMR6-LIKE OXYGENASE 2-like (A)	Protein DMR6-LIKE OXYGENASE 2 {ECO:0000303|PubMed:25376907}; EC=1.14.11.- {ECO:0000255|PROSITE-ProRule:PRU00805}; 2-oxoglutarate (2OG)-Fe(II) oxygenase-like protein DLO2 {ECO:0000303|PubMed:25376907}; Salicylate 3-hydroxylase DLO2 {ECO:0000305}; S3H DLO2 {ECO:0000305}; SA 3-hydroxylase DLO2 {ECO:0000305}; Salicylic acid 3-hydroxylase DLO2 {ECO:0000305}; EC=1.14.13.- {ECO:0000250|UniProtKB:Q9ZSA8} (At3g19000)		-1.274
Cs6g12050	K13430 serine/threonine-protein kinase PBS1 [EC:2.7.11.1] | (RefSeq) serine/threonine-protein kinase PBS1 (A)	Serine/threonine-protein kinase PCRK1 {ECO:0000305}; EC=2.7.11.1 {ECO:0000269|PubMed:25711411}; Protein PTI-COMPROMISED RECEPTOR-LIKE CYTOPLASMIC KINASE 1 {ECO:0000303|PubMed:25711411} (At3g09830)	1.726	
Cs7g27120	--	Protein SAR DEFICIENT 1 {ECO:0000303|PubMed:20921422} (At1g73800)	1.612	
Cs9g14480	K24028 salicylic acid 3-hydroxylase [EC:1.14.11.-] | (RefSeq)	Protein DMR6-LIKE OXYGENASE 2 {ECO:0000303|PubMed:25376907};	6.705	

	protein DMR6-LIKE OXYGENASE 2-like (A)	EC=1.14.11.- {ECO:0000255|PROSITE-ProRule:PRU00805}; 2-oxoglutarate (2OG)-Fe(II) oxygenase-like protein DLO2 {ECO:0000303|PubMed:25376907}; Salicylate 3-hydroxylase DLO2 {ECO:0000305}; S3H DLO2 {ECO:0000305}; SA 3-hydroxylase DLO2 {ECO:0000305}; Salicylic acid 3-hydroxylase DLO2 {ECO:0000305}; EC=1.14.13.- {ECO:0000250|UniProtKB:Q9ZSA8} (At4g10490)		
Cs9g14500	K24028 salicylic acid 3-hydroxylase [EC:1.14.11.-] | (RefSeq) protein DMR6-LIKE OXYGENASE 2-like (A)	Protein DMR6-LIKE OXYGENASE 2 {ECO:0000303|PubMed:25376907}; EC=1.14.11.- {ECO:0000255|PROSITE-ProRule:PRU00805}; 2-oxoglutarate (2OG)-Fe(II) oxygenase-like protein DLO2 {ECO:0000303|PubMed:25376907}; Salicylate 3-hydroxylase DLO2 {ECO:0000305}; S3H DLO2 {ECO:0000305}; SA 3-hydroxylase DLO2 {ECO:0000305}; Salicylic acid 3-hydroxylase DLO2 {ECO:0000305}; EC=1.14.13.- {ECO:0000250|UniProtKB:Q9ZSA8} (At4g10490)		1.351
novel.1617	--	Protein GRIM REAPER; Stigma-specific STIG1-like protein GRI; GRIp (At1g53130)		-2.523
novel.306	K13691 pathogen-inducible salicylic acid glucosyltransferase [EC:2.4.1.-] | (RefSeq) UDP-glycosyltransferase 74G1-like (A)	UDP-glycosyltransferase 74F2; EC=2.4.1.-; AtSGT1; Salicylic acid glucosyltransferase 1 (At2g43820)		1.729
orange1.1t01963	K24028 salicylic acid 3-hydroxylase [EC:1.14.11.-] | (RefSeq) protein DMR6-LIKE OXYGENASE 2-like (A)	Protein DMR6-LIKE OXYGENASE 2 {ECO:0000303|PubMed:25376907}; EC=1.14.11.- {ECO:0000255|PROSITE-ProRule:PRU00805}; 2-oxoglutarate (2OG)-Fe(II) oxygenase-like protein DLO2 {ECO:0000303|PubMed:25376907}; Salicylate 3-hydroxylase DLO2 {ECO:0000305}; S3H DLO2 {ECO:0000305}; SA 3-hydroxylase DLO2 {ECO:0000305};Salicylic acid 3-hydroxylase DLO2 {ECO:0000305}; EC=1.14.13.- {ECO:0000250|UniProtKB:Q9ZSA8} (At4g10490)	1.425	
